# BullFish: Software for an automated stepwise analysis of positional and postural kinematics of zebrafish locomotion

**DOI:** 10.1016/j.isci.2026.115798

**Published:** 2026-04-17

**Authors:** Sherry Sin-Hang Yeung, Ho-Ming Cheng, Chak-Kwan Lee, Wai-Hang Chan, Yong-qi Luo, Gordon Tin-Chun Wong, Raymond Chuen-Chung Chang

**Affiliations:** 1 Laboratory of Neurodegenerative Diseases, School of Biomedical Sciences, LKS Faculty of Medicine, The University of Hong Kong, Pokfulam, Hong Kong SAR, China; 2 Department of Anaesthesiology, LKS Faculty of Medicine, The University of Hong Kong, Pokfulam, Hong Kong Island, Hong Kong SAR, China

**Keywords:** neuroscience, behavioral neuroscience, techniques in neuroscience, biological sciences research methodologies

## Abstract

Zebrafish are a tractable vertebrate model for studying locomotor behavior across development. We applied BullFish, an automated tracking and gait-analysis tool that quantifies macro- and micro-parameters—speed, orientation, tail- and trunk-bend angles, body curvature, stroke symmetry, and postural changes—across ages, sizes, and arbitrary arena shapes. In targeted experiments using a dopaminergic lesion model (6-OHDA) and a fin-amputation model, BullFish binned instantaneous swimming speed and extracted kinematic metrics within each bin (tail-beat frequency and amplitude, burst duration, inter-bout interval, and turn velocity). Dopaminergic loss spared low-speed metrics but reduced high-speed tail-beat frequency and amplitude, shortened burst durations, and decreased high-acceleration bouts—implicating deficits in rapid propulsion. Fin amputation preserved high-speed bursts but altered low-speed control with fewer fine postural corrections—consistent with impaired low-force maneuvering and stability. BullFish also corrected common tracking errors from other tools and delivered consistent, stage-spanning locomotor phenotypes to improve behavioral interpretation in neurobiology and injury studies.

## Highlights

• BullFish performs stepwise locomotor assessment in zebrafish
• BullFish analyses gait asymmetries across various all developmental age
• BullFish uses highly accurate and self-correcting tracking algorithm
• BullFish identifies rapid movement dysfunction in 6-OHDA-induced Parkinson’s disease model

## Graphical abstract

**Figure:**
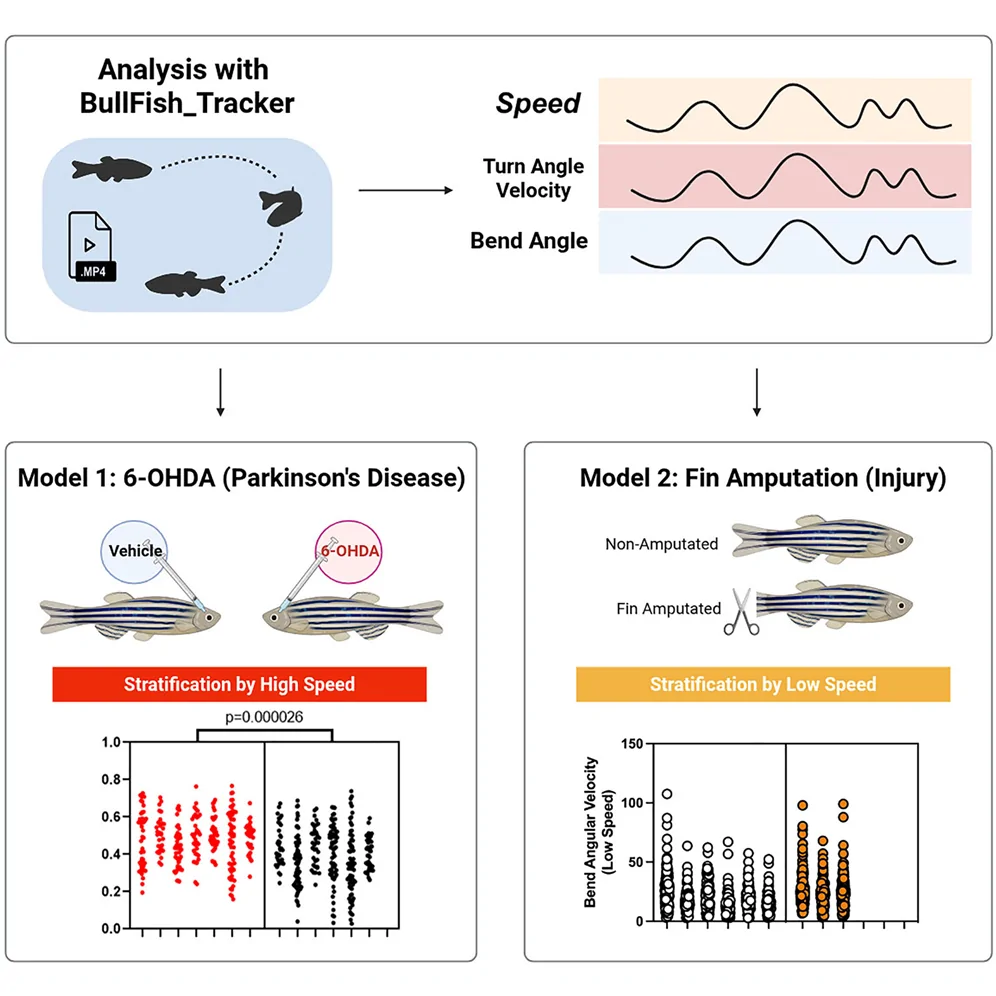

## Introduction

The zebrafish (*Danio rerio*) has emerged as a powerful model organism in neuroscience, primarily due to its impressive genetic homology with mammals, low experimental costs, and ease of genetic manipulation. This similarity allows researchers to investigate complex pathophysiological mechanisms with greater relevance to human health. As such, zebrafish have been increasingly utilized to model diseases like motor neuron degeneration (MND),^1^ Huntington’s disease,^2^ Tauopathies^3^ and Parkinson’s disease (PD),^4^ providing a unique platform for both basic research and translational studies.

Despite these attributes, the study of locomotion in zebrafish, particularly in relation to neurodegeneration, remains a significant challenge. While zebrafish locomotion is an essential aspect of motor function, existing analyses often oversimplify the complexities involved. Current locomotion assessments primarily focus on basic metrics such as swimming speed and distance traveled.^5^^,^^6^ These parameters, while useful, fail to encompass the intricate dynamics of locomotor behavior, especially in disease contexts. Gait analysis, which considers factors such as stride length, frequency, and symmetry, is largely underexplored in zebrafish research, yet it holds critical importance for understanding the nuances of motor dysfunction.

Interestingly, zebrafish gait exhibits similarities to human gait, particularly in how both species demonstrate compensatory movements in response to neurological impairments. For instance, just as humans with PD may display reduced stride length and altered walking patterns, zebrafish with dopaminergic deficits may show restricted turning angles and decreased agility.^5^^,^^7^^,^^8^ Quantifying these gait parameters could offer deeper insights into the motor deficits associated with neurodegenerative disorders, potentially mirroring the bradykinesia and rigidity seen clinically.^1^^,^^9^^,^^10^

Despite the burgeoning interest in zebrafish locomotion studies, existing methodologies often rely on subscription-based software and expensive, complex hardware, which may not be readily available to many laboratories due to financial or geographical constraints. This limitation severely hinders the widespread adoption of locomotion analysis in zebrafish research, even as the need for such studies continues to grow. The complexity and cost associated with current methodologies may deter researchers from conducting comprehensive locomotion analyses, ultimately impeding advancements in understanding neurodegenerative disorders.

To address this gap, we present the BullFish protocol, a straightforward and cost-effective setup designed to simultaneously characterize multiple parameters of zebrafish locomotion, including gait dynamics. BullFish stands for “*Behavioral Uncovering of Locomotion and Locomotor Parameters in Fish*,” and it offers a user-friendly platform that streamlines data collection and analysis. The protocol utilizes open-source software and standard video tracking technology, making it accessible to laboratories regardless of budget constraints. By integrating a range of locomotion metrics—such as swimming speed, distance, turning angles, and gait characteristics— BullFish provides a comprehensive view of zebrafish locomotor behavior.

The analysis parameters included in BullFish were identified through a systematic review of current locomotion paradigms in the literature. By offering a streamlined approach to locomotion analysis, the BullFish protocol aims to enhance reproducibility and accessibility in zebrafish studies. This initiative not only seeks to standardize the assessment of locomotion in zebrafish but also aspires to foster collaboration among researchers, ultimately accelerating discoveries in neuroscientific research. By making locomotion analysis more accessible, we aim to empower researchers to explore the intricate relationships between motor behavior and neurodegenerative diseases, thereby advancing our understanding of these complex conditions.

## Results

### The locomotion of zebrafish across all developmental stages is a focal point of research in various scientific fields

Conducting a systematic review of the literature is essential in evaluating the necessity of the BullFish protocol for characterizing zebrafish locomotion. To this end, 86 independent studies were isolated under PRISMA regulations (Figure S1), after vigorous reviews of the inclusion and exclusion criteria listed in the methodology. Within the analysis, our results demonstrated that fields such as pharmacology (15.1%), neuroscience (30%), and toxicology (25.6%) had the highest use of zebrafish locomotion. In combination, these fields account for approximately 70% of all the screened studies in the systematic review (Figure S2A), demonstrating a significant need for zebrafish locomotion analysis in biological research.

It has been reported that there are inter-strain variations in zebrafish locomotor ontogeny. Some common strains in zebrafish include wild types AB, TU, TL, SF, Golden, Turku, and Casper—a genetically modified strain with translucent phenotypes through disruption of specific pigmentation genes (e.g., tyr, mitfa, ednrb1, and sox10). For instance, studies have shown that Casper strains have reduced swimming mobility times compared to the AB strain. Of the reviewed studies, approximately 51% did not report the strain of zebrafish used despite obvious differences in locomotion (Figure S2C). Of the reported studies, approximately 23.2% of locomotion studies used the AB strain.

A large number of studies (93%) performed locomotion analysis on both wild type and transgenic strains (Figure S2B), indicating the diverse application of locomotor analysis. However, more than half of these studies did not mention the weight or length of the zebrafish, which might affect locomotion in aspects such as speed and turning (Figure S2E). Of the reported studies, the analysis of locomotion is mostly performed in zebrafish aged 4–6 months post-fertilization (Figure S2F), when swimming versatility is most robust. Sex differences for locomotion were not accounted for, as most studies adopted randomized sex sampling strategies (Figure S2G).

### Hardware considerations are often underreported within the literature

To design the appropriate hardware for locomotion analysis, we examined the most used shapes for the behavior chamber. Of the 86 independent studies, approximately 47.6% did not report the shape of the behavior chamber. Of the reported studies, behavior chambers adopted a diverse range of shapes, including trapezoidal (standard zebrafish storage tanks), cone, cuboidal, tubular, pentagonal, cylindrical, and most commonly used—the rectangular tank (approximately 31.4%) (Figure S3A). Other important hardware considerations included the volumetric load (Figure S2B) and whether the locomotion analysis was performed in a closed chamber environment (Figure S2C). Both the camera brand and the required resolution were crucial aspects of the hardware setup; however, approximately 64.7% and 78.9% of the studies did not provide this information (Figures S3D and S3E).

In other considerations, zebrafish present varying locomotive responses depending on their circadian rhythms. The literature heavily discusses changes in locomotion patterns depending on day or nighttime exploratory behavior. In general, daytime exploration includes increased activity levels with higher swimming speeds, while nighttime exploration is limited to moments of decreased swimming bouts and increased erratic swimming patterns. Nevertheless, we extracted studies that reported experimental time and concluded that most locomotion was examined from 1,000 to 1,600 (Figure S3F).

### ANY-maze and Ethovision are the largest players in zebrafish locomotion analysis

Among the eligible studies in this systematic review, we categorized the various locomotion analysis platforms utilized. The platforms included ANY-maze, Ethovision, idTracker, ImageJ, Viewpoint Life Science, Viewpoint Behavior Technology, ToxTrac, Zebrafish Tower System, Viewpoint Zebrafish Lab, DeepLabCut, ZebTrack, Ctrax (Python), and several in-house developed codes. Notably, the most frequently employed platforms were ANY-maze (*n* = 20) and Ethovision (*n* = 25), which together represented approximately 52.3% of the studies analyzed (Figure S4A).

### Zebrafish locomotion can be analyzed by a variety of parameters, where some were better represented than others within the literature

Zebrafish locomotion can be analyzed through a variety of parameters. Through our systematic review, we have isolated 11 parameters that were discussed when zebrafish locomotion was analyzed. Of these, (1) freezing time, (2) swimming speed, and (3) total distance traveled were most significantly reported. 81.4% of studies utilized “swimming speed” as a locomotive assessment, making it the most frequently discussed. Following so, 48.8% of studies analyzed “freezing time”, and 36.1% reported “total distance traveled” (Figure S4D). These parameters are the easiest to obtain in terms of recording and software analysis, while being sensitive to many conditions. However, parameters of more specific aspects of locomotion such as gait and tail positions were not discussed. Only approximately 10%–25% of the studies evaluated these parameters, which is understandable because evaluating these parameters requires high-speed recording and complex computational algorithms. BullFish, as illustrated later, aims to make these parameters simpler to analyze. We noted that 61.62% of these studies did not report the frame rate used for locomotion analysis (Figure S4B). Moreover, 72.7% of the reported studies used a frame rate of only 10–30 fps. Only 27.3% of the reported studies used frame rates of 60 fps or above for complex locomotion analysis (Figure S4B). Generally, a higher frame rate of approximately 60–120 fps is required for 3D locomotion analysis. In our systematic review, only 18.5% of studies used 3D analysis, while 81.3% performed 2D analysis (Figure S4C). 3D analysis provides more comprehensive information on zebrafish locomotion as zebrafish also exhibit a variety of behavior on the *z* axis, such as diving, although it is also more complicated to record locomotion in 3D in aspects such as recording, synchronization, animal tracking, and computation.

### BullFish software automatically tracks the position and posture of zebrafish

While previous studies involving analysis of zebrafish locomotion have established the role of locomotion analysis in research using zebrafish models, current methods remain rudimentary compared to gait analysis in mammalian models and humans. The difference is that gait analysis in these subjects measures not only the speed and distance (linear and rotational) achieved by the subject, but also the changes in posture in terms of joint angles, oscillations, symmetry, variability, etc. Zebrafish similarly can be analyzed in these aspects as they move from one place to another by episodes of body undulations. However, as illustrated previously, tail bend angles and frequencies were rarely measured. The reason might be the technical challenge of calculating them accurately without human intervention, but this problem is directly addressed by the BullFish protocol. We developed BullFish with the aim of allowing free, convenient yet comprehensive analysis of locomotion of adult zebrafish.

**Figure 1:**
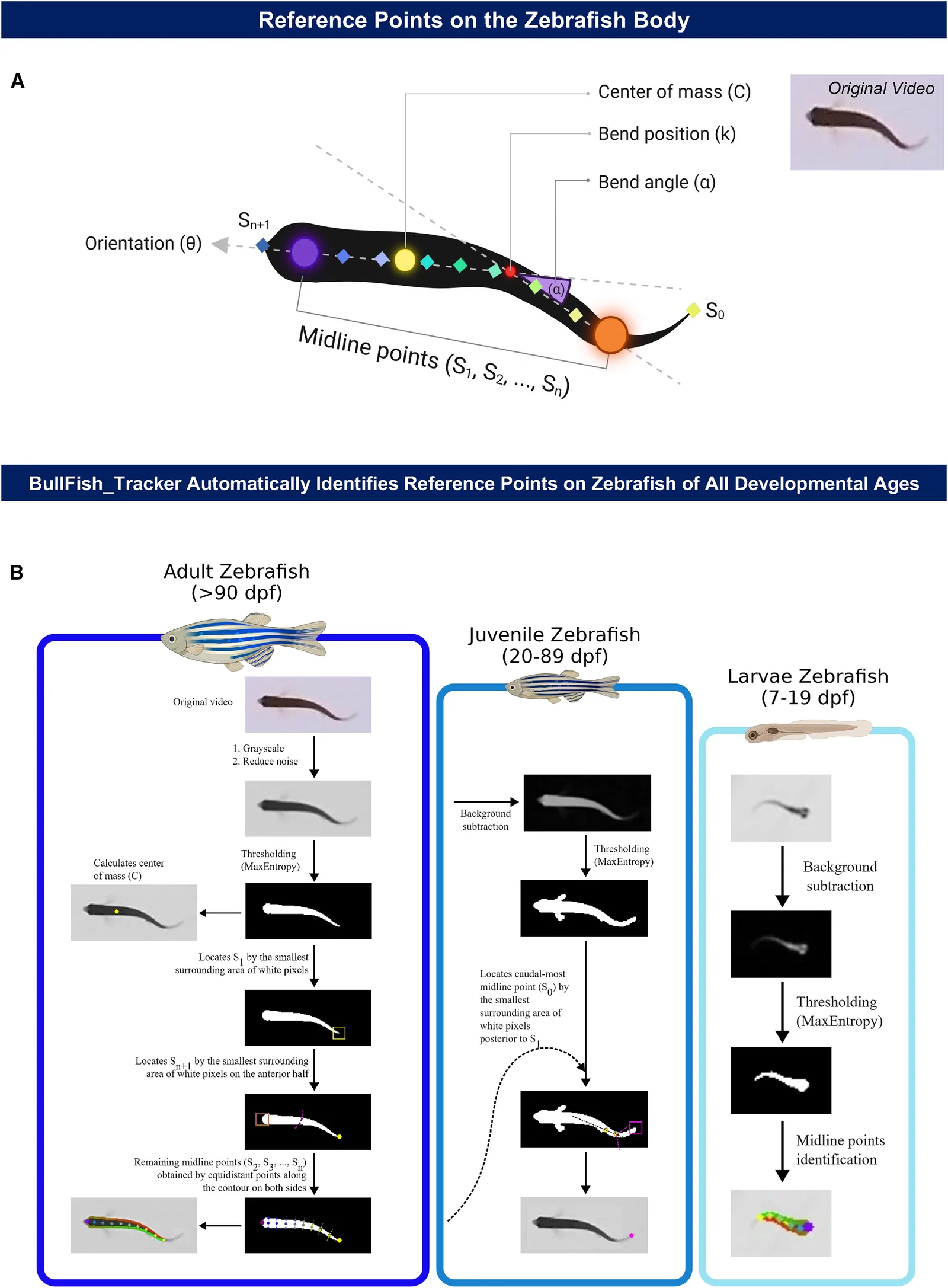
BullFish is a streamlined protocol that evaluates zebrafish locomotion across various developmental ages and sizes (A) BullFish automatically profiles the position and posture of the zebrafish. An illustration of the points representing the position (center of mass) and posture (midline points) of the zebrafish, as defined in BullFish. Orientation, bend angle, and bend position are variables calculated from the points. (B) An illustration of the streamline workflow for zebrafish locomotion using BullFish. In brief, zebrafish locomotion is captured by video and fed into BullFish_Tracker. First, grayscale is performed, followed by thresholding (MaxEntropy). This ensures the maximum reduction of noise and artifacts during video capture. BullFish calculates and recognizes the center of mass, c, denoted by yellow dot. Following so, S1 (tail) is located the smallest surrounding area of white pixels. Various Sn+1 points along the midline are obtained by equidistant dots along the counter of both the asymmetrical streamline zebrafish body. BullFish can be automatically adapted to perform locomotion analysis of zebrafish in different developmental ages and sizes. Juvenile zebrafish (approximately 20–89 dpf) and larvae (approximately 7–19 dpf) adopted similar thresholding and tracking techniques as fully developed zebrafish adults, but with higher precision.

To describe the position and posture of zebrafish, a set of points are defined at the top view of zebrafish (Figure 1A). Its position is represented by the center of mass (C). Its posture is represented by a number of points along the midline from caudal to cranial (S_1_,S_2_,S_3_,⋯,S_n_). The number of points (n) is determined by the lengths of the group of zebrafish under testing—we set it as 10 for large adults, 7 for small adults, 6 for juveniles, and 5 for larvae. They allow quantification of the degree of body bending. S_1_ starts at around the base of the caudal fin, and with S_2_ forms a line that is tangent to the distal-most part of the midline. S_n_ and S_n-1_ forms a line that is tangent to the head midline, and the angle (α) between these two lines describes the degree of bending. Since zebrafish undulation occurs as a wave traveling from proximal to distal,^11^ to describe the zebrafish posture more completely, bend position (k) is defined as the position on the midline where the distal part begins to be laterally flexed. It is represented by a value, 0 to 1, that is the ratio of the length from tail to bend position to the whole fish length. Finally, the orientation of the zebrafish (θ) is represented by the line formed by S_n_ and S_n-1_, and it allows quantification of the degree of turning movements. Detailed calculations of the four quantities representing position and posture (speed (calculated from C), orientation, bend angle, bend position) are shown in Table S1.

To track the changes in position and posture, the coordinates of these points are obtained in every frame of the video by BullFish. BullFish consists of three main programs that work together seamlessly. The first program, “BullFish_videosetup”, is responsible for gathering user inputs. It prompts users to specify the video segment for analysis, define the cropping area, and isolate the swimming area for tracking. This setup ensures that the subsequent analysis is relevant and the raw data generated is systematically stored. The second program, “BullFish_tracker”, operates automatically to process the selected videos and extract the crucial coordinates mentioned previously (Figure 1B).

In detail, after conversion to grayscale and Gaussian blurring for noise reduction, the video is binarized using the maximum entropy algorithm (referenced from an ImageJ function) to differentiate between the dark zebrafish and the light background. This contrast allows for accurate detection of the zebrafish contour. The overall location of the zebrafish is represented by its center of mass (C), obtained from formula. Then, BullFish_tracker identifies the tail (S_1_) as the sharpest point by analyzing each contour pixel and its surrounding area. It applies a square to each pixel to calculate the white area within it. Since the tail is the thinnest part of the zebrafish, it exhibits the smallest white area, making it easily identifiable. Proximal pixels are at the wider part of the body and therefore surrounded by larger white areas, which helps in distinguishing the tail from the rest of the body. The head, on the other end of the body, also has contour pixels with smaller surrounding white areas compared to the trunk. Thus, the sharpest point on the proximal half of the body is designated as the head (S_n+1_). Following this, the contour is split into two-halves, and each half is divided into 4, 5, 6, or 9 equidistant points, depending on the size of zebrafish. By connecting the pairs of equidistant points and obtaining the respective midpoint, the remaining midline points (S_2_,S_3_,⋯,S_n_) are derived, representing the zebrafish posture. These points together provide a comprehensive view of zebrafish locomotion.

This process is used in zebrafish adults. Of note, background subtraction is deliberately not performed, unlike other tracking software.^12^^,^^13^ This is to allow the binarization to remove the pectoral fins, which are light in color as opposed to the body. If background subtraction is performed, the pectoral fins will show up after binarization, which will interfere with midline points identification, a common problem in zebrafish tracking.^14^ However, for smaller zebrafish, the tail is less dark and consequently, binarization without background subtraction will erode the zebrafish contour excessively, preventing the program from recognizing subtle bending movements. To address both problems, binarizations with and without background subtraction are performed in parallel in smaller zebrafish like juveniles. Binarization without background subtraction is followed by the same process of reference points identification. The S_1_ here will be too proximal, so a more distal point, S_0_, is identified from the tail of the contour obtained from binarization with background subtraction. The contour isolated in this process retains the lighter parts of the body. The pectoral fins will not be identified as S_0_ because S_1_ allows the program to exclude contour points proximal to S_1_.

For zebrafish larvae, binarization without background subtraction is difficult because of the light body color. Therefore, the process of contour identification follows the conventional steps of background subtraction and binarization. The process of reference points identification is same as that in adults.

### BullFish automatically identifies and corrects tracking errors

**Figure 2:**
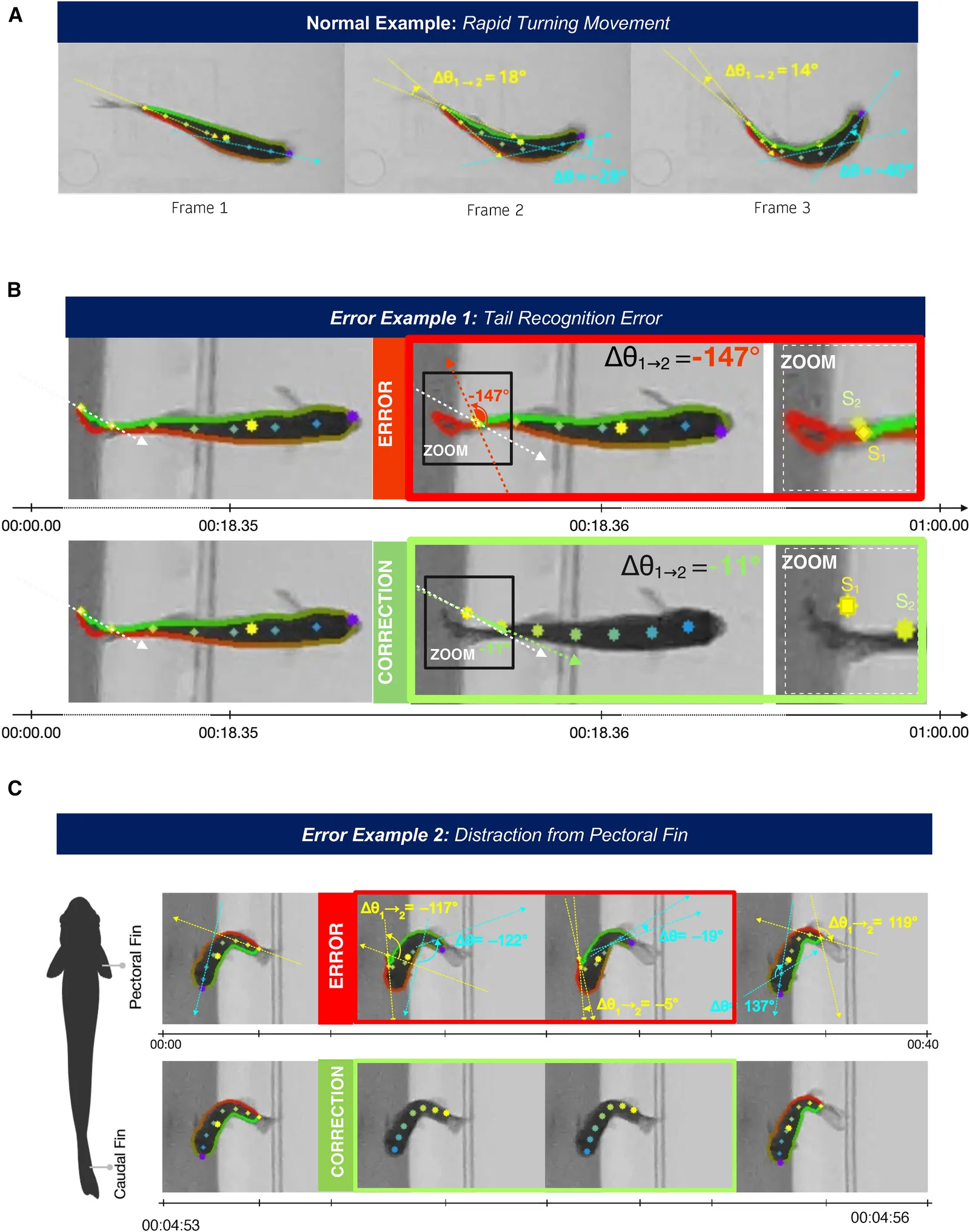
BullFish automatically identifies and corrects recognition errors during complex zebrafish locomotion maneuvers (A) During “rapid turning movement”, zebrafish turns its body such that the thinnest point (i.e., smallest surrounding area of white pixels, S_1_) may be incorrectly located. Here, frames 1, 2, and 3, denote an example of correct point recognition. (B) Frame 2 denotes an example of tail recognition error. In the error (red) panel, points S_1_ (yellow dot) and S_2_ (green dot) are wrongly recognized and converge on the same point. This creates an incorrect radial calculation of −147°. BullFish has the capability to automatically correct this error, as shown on the correction (green) panel. Here, points S_1_ (yellow dot) and S_2_ (green dot) correctly recognizes the end of the zebrafish tail and its equidistant midline points, and showed the correct radial calculation of −11°. (C) Another common recognition error is caused by a distraction from the pectoral fin during extreme turning events in zebrafish locomotion. The error panel (red) shows a misplacement of point S_1_ (yellow dot) in the trunk of the fish. BullFish automatically detects this as shown in the correction panel (green), where the S_1_ (yellow dot) was correctly placed near the caudal fin.

To allow the researcher to verify the accuracy of tracking of BullFish_tracker, it produces a video with the reference points annotated on the zebrafish (Figure 2) so that the researcher can look for any point misplacements. S_1_ is the yellow point at the tail, and going proximally the midline points change from yellow to blue. The zebrafish contour is highlighted in red on one side, transitioning to green on the other side. The center of mass (C) is denoted by the larger yellow circle at the center. However, manual inspection is time-consuming as the video has to be played at slower speeds to avoid missing errors.

The third program of BullFish, “BullFish_analysis”, automatically identifies and corrects tracking errors. Errors result from the fins (Figures 2B and 2C) show the interference from caudal and pectoral fins, respectively. They cause misplacement of S_1_ because they are also at the thinner parts of the body. These errors are rare because our method of binarization usually excludes the fins, but they nonetheless occur in single or a few consecutive frames. To automatically identify these errors, BullFish_analysis calculates the orientations of S_1_S_2_ ($\theta_{1\to 2}$) and S_n-1_S_n_ (θ), respectively, in each frame and traces their changes (${\Delta \theta }_{1\to 2}$ and Δθ) across each frame. Normally, ${\Delta \theta }_{1\to 2}$ and Δθ can reach the magnitudes 18° and 40°, respectively, across one frame, i.e., 0.01 s, in an episode of rapid turning movement (Figure 2A). In contrast, misplacing S_1_ proximal to the tail causes S_2_ to be misplaced distally, resulting in a ${\Delta \theta }_{1\to 2}$ of magnitude 147° while Δθ is reasonable (Figure 2B). In Figure 2C, misplacing S_1_ at the right pectoral fin disrupts all the midline points. ${\Delta \theta }_{1\to 2}$ and Δθ are −117° and −122° when the misplacement occurs at the second frame, −5° and −19° when the misplacement continues at the third frame, 119° and 137° when correct placement resumes. Hence, there is a threshold value for ${\Delta \theta }_{1\to 2}$ and Δθ such that it is larger than that of normal rapid movements but smaller than that of appearance and resolution of point misplacements. We set this value as 57°, which is 17° larger than the quickest movement we observed. With this criterion, BullFish_analysis automatically identifies frames where point misplacement appears or resolves. The midline points of each frame in between are replaced by points predicted from the starting and ending frames (Figures 2B and 2C). Of note, after identifying an appearance of point misplacement, if the program cannot identify the resolution of point misplacement within the next 5 frames, this suspected series of errors will not be corrected in order to avoid overcorrection. However, errors lasting this long will be obvious to the researcher, and we did not observe such errors when using BullFish.

### BullFish is highly accurate in tracking zebrafish across all developmental stages

**Figure 3:**
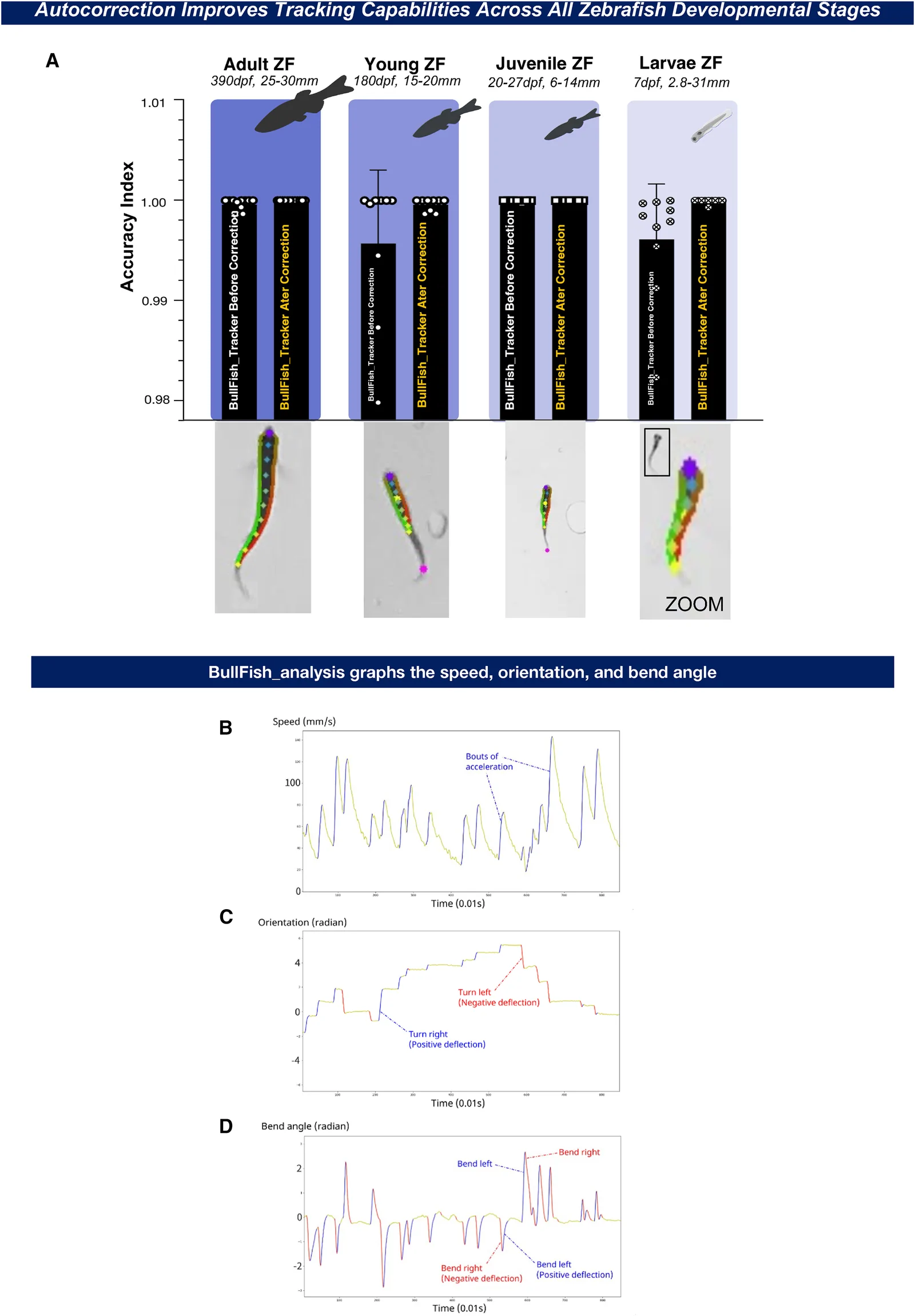
Autocorrection allows BullFish to quantify locomotion for zebrafish (ZF) of various developmental size and ages (A) Bar chart comparing the accuracy index of BullFish_Tracker before (left panel) and after correction (right panel) in Adult ZF (390 dpf, 25–30 mm), Young ZF (180 dpf, 15–20 mm), Juvenile ZF (20–27 dpf, 6–14 mm), and larvae (7 dpf, 2.8–3.1 mm). (B–D) Representative images of zebrafish of various development size and age and BullFish points of reference are shown in the lower panels (underneath the bar chart). BullFish_analysis produces a graph on (B) speed (mm/s), (C) orientation (radian), (D) bend angle (radian).

With the capability of automatic error detection and correction, we assessed the accuracy of tracking of BullFish in zebrafish of different developmental stages (Figure 3A). It is measured by the accuracy index, which is the number of error frames detected by BullFish_analysis divided by the total number of frames of the video segment. Before correction, the mean accuracy indices were already 0.9996 (range: 0.9985–1.0000) and 1.0000 (range: 0.9997–1.0000) for large adult zebrafish (390 dpf, 25–30 mm) and juveniles (20–27 dpf, 6–14 mm), respectively. Tracking of smaller adult zebrafish (180 dpf, 15–20 mm) and larvae (7 dpf, 2.8–3.1 mm) was also reasonably accurate before correction, with mean accuracy indices being 0.9957 (range: 0.9798–1.0000) and 0.9961 (range: 0.9824–1.0000), respectively. After correction, there were no errors detected for large adult zebrafish and juveniles, i.e., accuracy index = 1.0000. The mean accuracy indices for smaller adult zebrafish and larvae were 0.9996 (range: 0.9987–1.0000) and 0.9999 (range: 0.9993–1.0000) after correction. This shows the high accuracy of tracking of BullFish, which is crucial for analyzing subtle movements of zebrafish.

### BullFish reveals episodic movements of zebrafish

As mentioned, the reference points of zebrafish derive four quantities describing the position and posture of zebrafish—speed (v), orientation (θ), bend angle (α), and bend position (k). Plotting them against time (Figure 3B) reveals the pattern of zebrafish locomotion. Within the 8 s, speed varies greatly in the form of peaks of different sizes, instead of being a smooth curve. Each peak represents an episode of acceleration followed by deceleration. Orientation also changes in episodes, each representing a left (negative) or right (positive) turn. It remains constant in between. Bend angle also changes in episodes, each representing a left (positive) or right (negative) tail bend. They usually occur in a series of two to four consecutive tail bends, with the first tail bend deviating from neutral position and the last returning to neutral position. Bend position is not plotted here because its interpretation is only meaningful within an episode of tail bend.

### BullFish restructures the analysis of locomotion into macro- and micro-parameters

With the observation that zebrafish swim in episodes of exertion that bring them from one place to another, analysis of their locomotion can be divided into macro-parameters describing the overall result, and micro-parameters describing individual episodes of swimming.

Macro-parameters are listed in Table S2. They include commonly measured parameters such as distance traveled, freezing, active speed, maximum speed, and thigmotaxis, and statistic aggregates of micro-parameters (to be elaborated later).

**Figure 4:**
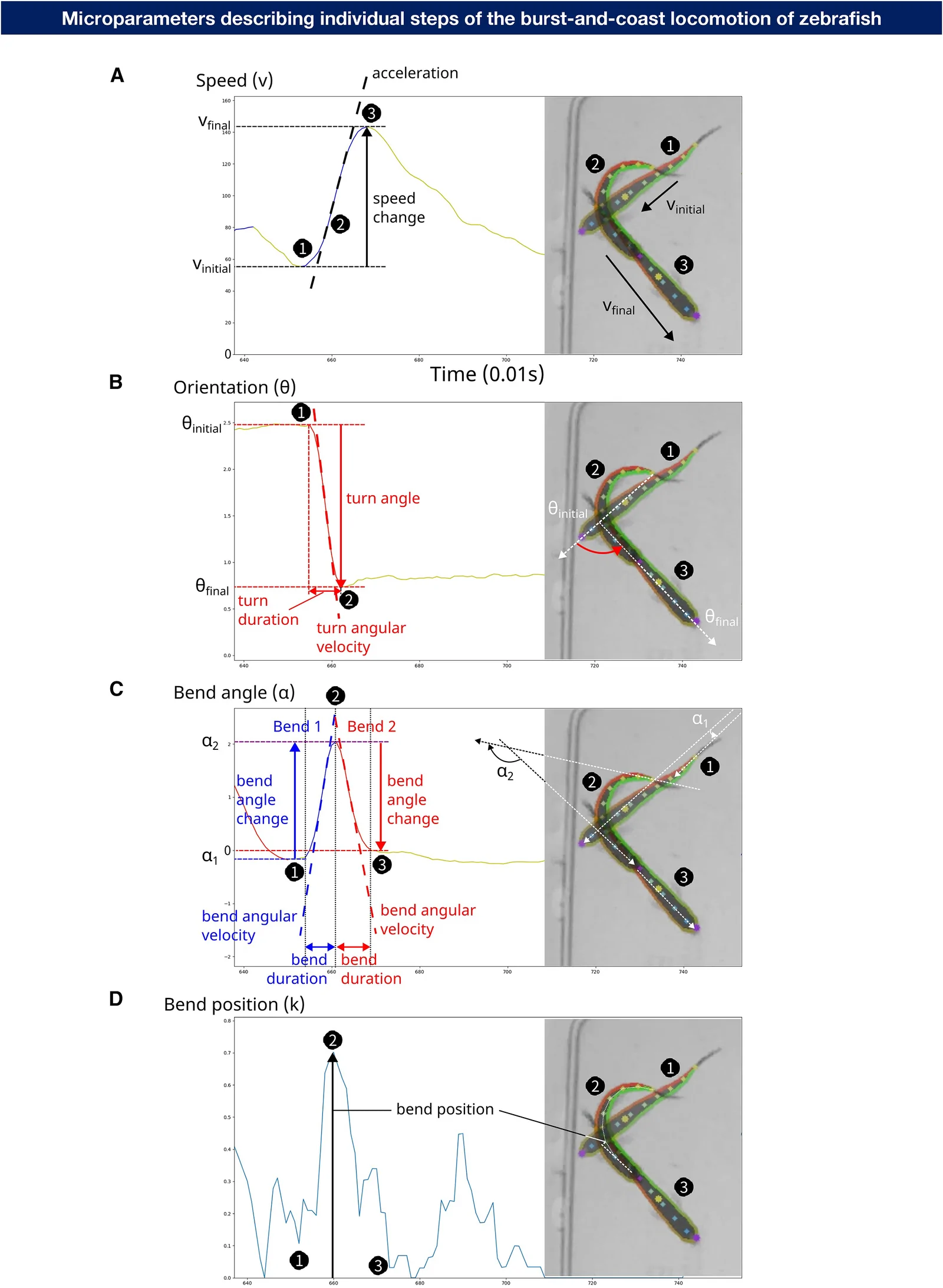
Micro-parameters of individual episodes of accelerations, turns, and tail bends (A) Speed-time (v-t) graph of a short video segment. The picture on the right shows the zebrafish at three time points (1, 2, and 3) overlayed together, and they are annotated at the corresponding positions of the graph. At time point 1, the zebrafish starts to accelerate, continuing until near time point 3. The black dotted line is tangent to the upslope of v, and its slope represents the value of acceleration. The speed change in this acceleration equals v_final_-v_initial_. (B) Orientation-time (θ-t) graph of the same video segment. At time point 1, the zebrafish starts to turn left, and stops turning at time point 2. Similarly, the slope of the red dotted line represents the turn angular velocity and the turn angle equals θ_final_-θ_initial_. Turn duration is also illustrated on the graph. The picture on the right is same as that in (A), and θ_initial_ and θ_final_ are annotated by white arrows. Turn angle is illustrated by the red arrow. (C) Bend angle-time (α-t) graph. At time point 1, the zebrafish starts to bend left, and starts to bend right at time point 2, returning to neutral position at time point 3. Similarly, the slope of each dotted line represents the bend angular velocity of that tail bend. Bend angle change equals α_2_-α_1_ for the first tail bend, where α_1_ and α_2_ are the bend angles at time points 1 and 2, respectively, as annotated on the right picture. α is 0 at time point 3 in this example, so the bend angle change of the second tail bend is 0-α_2_. Bend duration of each tail bend is shown on the graph. (D) Bend position-time (k-t) graph. The zebrafish is maximumly flexed at time point 2, so the bend position for both tail bends is measured here as shown on the graph. The right figure illustrates that the zebrafish begins to be flexed at S_7_, meaning that k ≈ 6/8. (There are nine midline points in this illustration; 10 are used for the actual analysis).

Micro-parameters are listed in Table S3. Each episode of acceleration, turn, and tail bend can be characterized by a number of micro-parameters as illustrated by a short video segment in Figure 4. In this video segment, three time points (1, 2, and 3) are captured and overlayed on the same photo. The zebrafish undergoes one episode of acceleration (top graph), with the slope of the black dotted line tangent to the upslope representing acceleration and the difference between peak (final) speed and trough (initial) speed representing speed change. Within the same time, the zebrafish makes a left turn (second-top graph), which can be characterized by its turn angle (θ_final_-θ_initial_), turn angular velocity (slope of tangent), and turn duration. Also happening concurrently is that the zebrafish makes a left tail bend followed by a right tail bend (second-bottom graph). Each tail bend can be characterized by bend angle change (α_2_-α_1_ for the first example, 0-α_2_ for the second example), bend angular velocity (slope of tangent), bend position (the maximum bend position within the bend duration, shown in the bottom graph), and bend duration.

Each zebrafish undergoes hundreds of episodes of movements in 1 min. They can be compared among groups of zebrafish using linear mixed models, and can be further stratified by different contexts for focused comparison, as illustrated in subsequent sections. They can also be statistically aggregated to give macro-parameters that give an overall impression of the zebrafish (Table S2). They include turn count, total turn angle, total turn duration, bend count, total bend angle, total bend duration, and step count. “Step” will be introduced next. Furthermore, BullFish can identify the direction of each turn and tail bend, enabling quantification of left-right asymmetry.

### Zebrafish swim in steps, representing the burst-and-coast behavior

**Figure 5:**
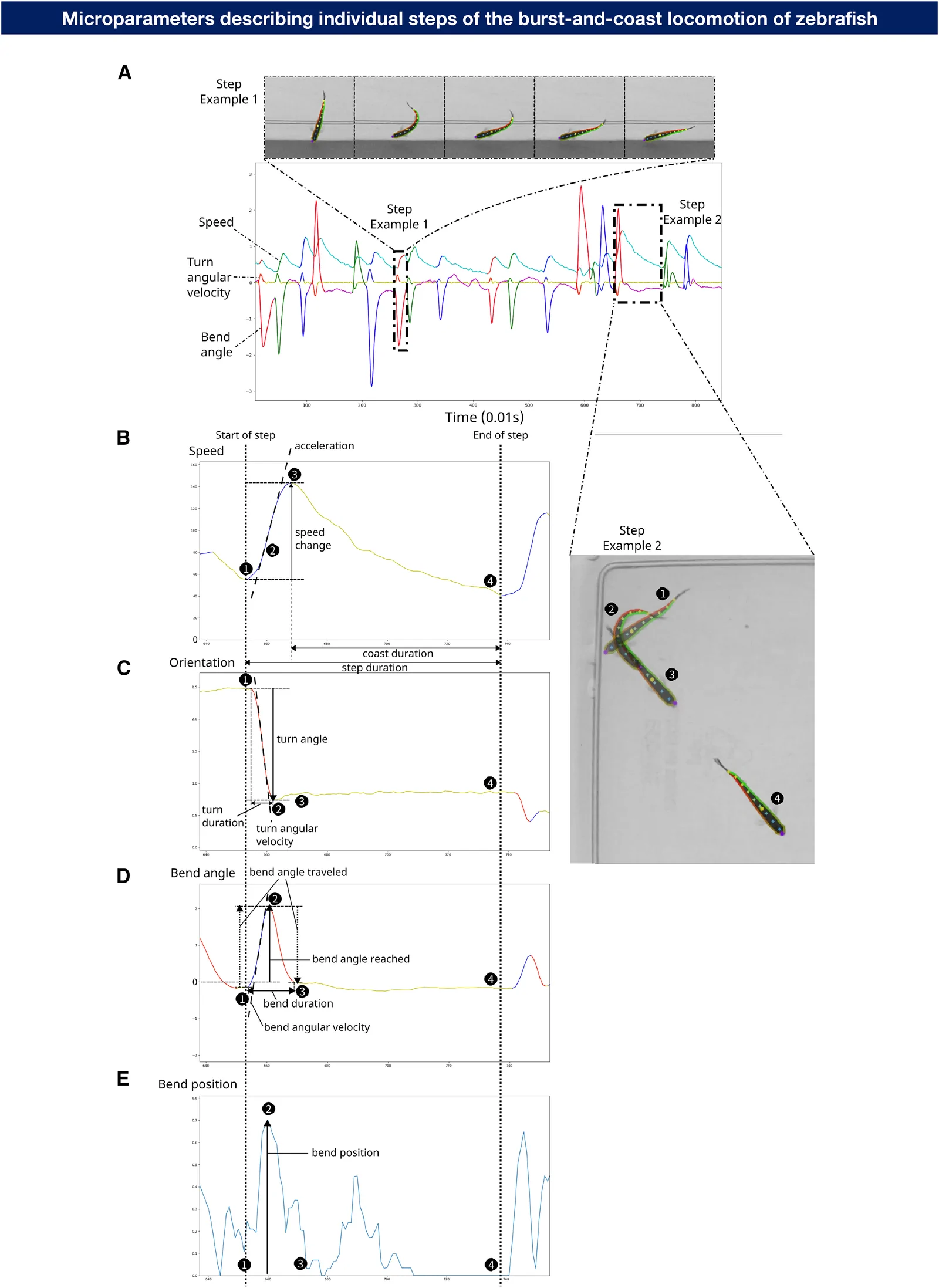
Micro-parameters describing individual steps of the burst-and-coast locomotion of zebrafish (A) Speed (cyan), turn angular velocity (yellow), and bend angle (magenta) are plotted against time together. They are highlighted in groups, with colors cycling from red, green, to blue. Each group has speed, turn angular velocity, and bend angle highlighted in the same color, constituting a step. Two examples of a step are selected using black dotted-line boxes. In example 1, the zebrafish makes a right tail bend followed by a left tail bend, bringing about a right turn and an acceleration. Example 2 is explained in four time points. (B) Speed-time (v-t) graph of the same video segment. The acceleration of example 2 is illustrated by the slope of the black dotted line tangent to the upslope of v, and speed change is the change of v from time point 1 to 3. The zebrafish undergoes coasting from time point 3 to 4, and the coast duration and step duration are also illustrated. (C) Orientation-time (θ-t) graph. The micro-parameters are the same as those of turns. (D) Bend angle-time (α-t) graph. The zebrafish undergoes left tail bend and then right tail bend. The bend angle reached of example 2 is the magnitude of α at time point 2, when the zebrafish is most flexed. Bend angle traveled is the sum of the magnitudes of the change of α from 1 to 2 and 2 to 3. Bend duration is from 1 to 3. Bend angular velocity is the maximum magnitude of slope of α from 1 to 3, and is represented by the black dotted line here. (E) Bend position-time (k-t) graph.

By examining the time courses of accelerations, turns, and tail bends, it can be observed that they occur in close succession. For instance, tail bends bring about an acceleration and/or a turn in a step (Figure 5). At the top graph, speed, turn angular velocity, and bend angle are plotted against time together. They are highlighted in groups, with colors cycling from red, green, to blue. Each group has speed, turn angular velocity, and bend angle highlighted in the same color, constituting a step. (Turn angular velocity is plotted here instead of orientation for easier illustration.) Two examples of a step are selected using black dotted-line boxes. In example 1, the zebrafish makes a right tail bend followed by a left tail bend, bringing about a right turn and an acceleration. Example 2 shows the same movement episodes in Figure 4. From time point 1 to 2, the zebrafish makes a left tail bend, bringing about a left turn and an acceleration. From time point 2 to 3, the zebrafish makes a right tail bend, but without any turn. This right tail bend can be regarded as obligatory as the tail has to return to neutral position after the body makes a left turn. Acceleration continues until the right tail bend almost finishes. Time point 1 to 3 represents the burst phase, while time point 3 to 4 represents the coast phase, in which the zebrafish makes no active movement and glides forward with the momentum acquired from the tail bend.

Each step can be characterized by a number of micro-parameters (Table S3). First, it includes the micro-parameters of acceleration and turn, as each step contains an acceleration and/or a turn. For bend angle, the micro-parameters are different because one step contains at least two tail bends. To summarize the features of the series of tail bends, bend angle reached is the maximum bend angle attained in a step; bend angle traveled is the sum of bend angle change of all tail bends; bend angular velocity is the maximum bend angular velocity of all tail bends; bend duration (total) is the sum of bend duration of all tail bends; bend wave frequency is the number of tail bends divided by the bend duration (total). In addition, to describe the burst-and-coast locomotion of zebrafish, coast duration (Time point 3 to 4), step duration (Time point 1 to 4), coast percent (proportion of step duration that is coast duration), and step length (distance traveled within the step duration; similar to stride length in humans) are calculated.

### BullFish is more accurate than similar tracking software

**Figure 6:**
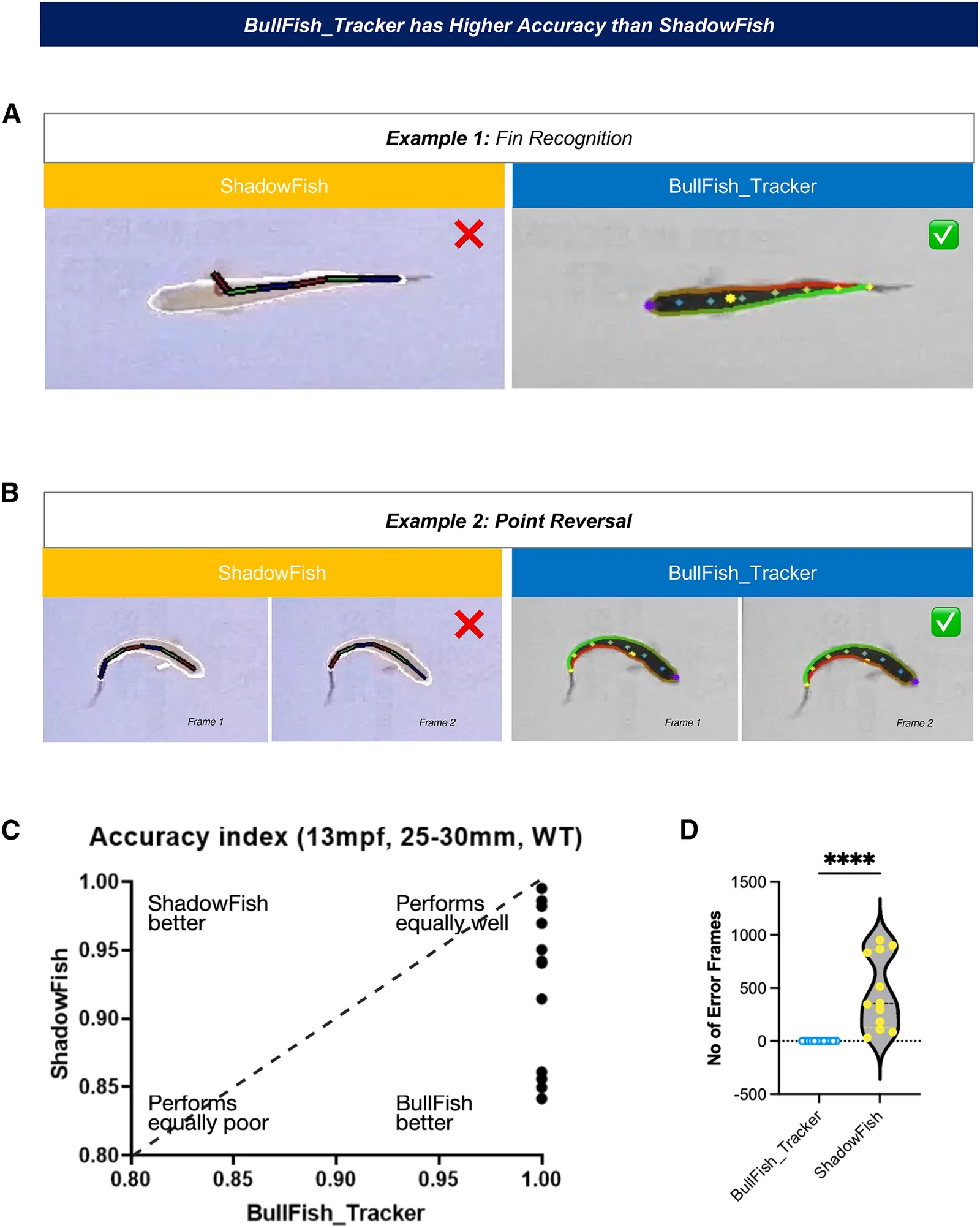
BullFish has a higher recognition accuracy compared to Shadowfish (A) Errors in fin recognition occurs in Shadowfish (yellow, left image), but is not made in BullFish_Tracker (blue, right image). (B) Errors in point reversal often occur in Shadowfish (yellow, left image, frame 2), but is not made in BullFish_Tracker (blue, right image, frame 2). (C) Graph plotted of the accuracy index between Shadowfish (*y* axis) and BullFish_Tracker (*x* axis), where BullFish has a higher accuracy index than Shadowfish. (D) Shadowfish has a higher number of error frames compared to BullFish_Tracker. Data were analyzed for statistical significance using Mann-Whitney U (for two-group comparisons), unless otherwise stated. All data are presented as mean ± SEM, where *p* < 0.05, ∗; *p* < 0.01, ∗∗; *p* < 0.001, ∗∗∗, where 12 individual zebrafish locomotion videos were analyzed.

To further ensure the accuracy of BullFish, we compared it with ShadowFish,^12^ one of the few tracking software that can obtain bend angles of adult zebrafish. It converts the midline into seven points, so we set *n* = 7 for the BullFish midline points. The input consisted of 13 videos of adult zebrafish, but one of them failed to be processed by ShadowFish despite choosing several different video segments and backgrounds. ShadowFish produced tracked videos with annotations of the zebrafish midline, and on manual inspection, misplacement of the midline often occurred, mainly deviating to the pectoral fins (Figure 6A). Closer examination can further reveal that ShadowFish did not distinguish between the head and tail, so the midline points often reversed its order (Figure 6B). Using the accuracy index described previously, we compared the accuracy of tracking of the 12 videos of the two softwares (Figure 6C). BullFish achieved full accuracy (Accuracy index = 1) for all videos, while ShadowFish achieved a mean accuracy index of 0.9240 (range: 0.8497–0.9950). In terms of the number of error frames (Figure 6D), BullFish did not have any, while ShadowFish had a mean of 456.2 error frames in a 6000-frame video (range: 30–951).

**Figure 7:**
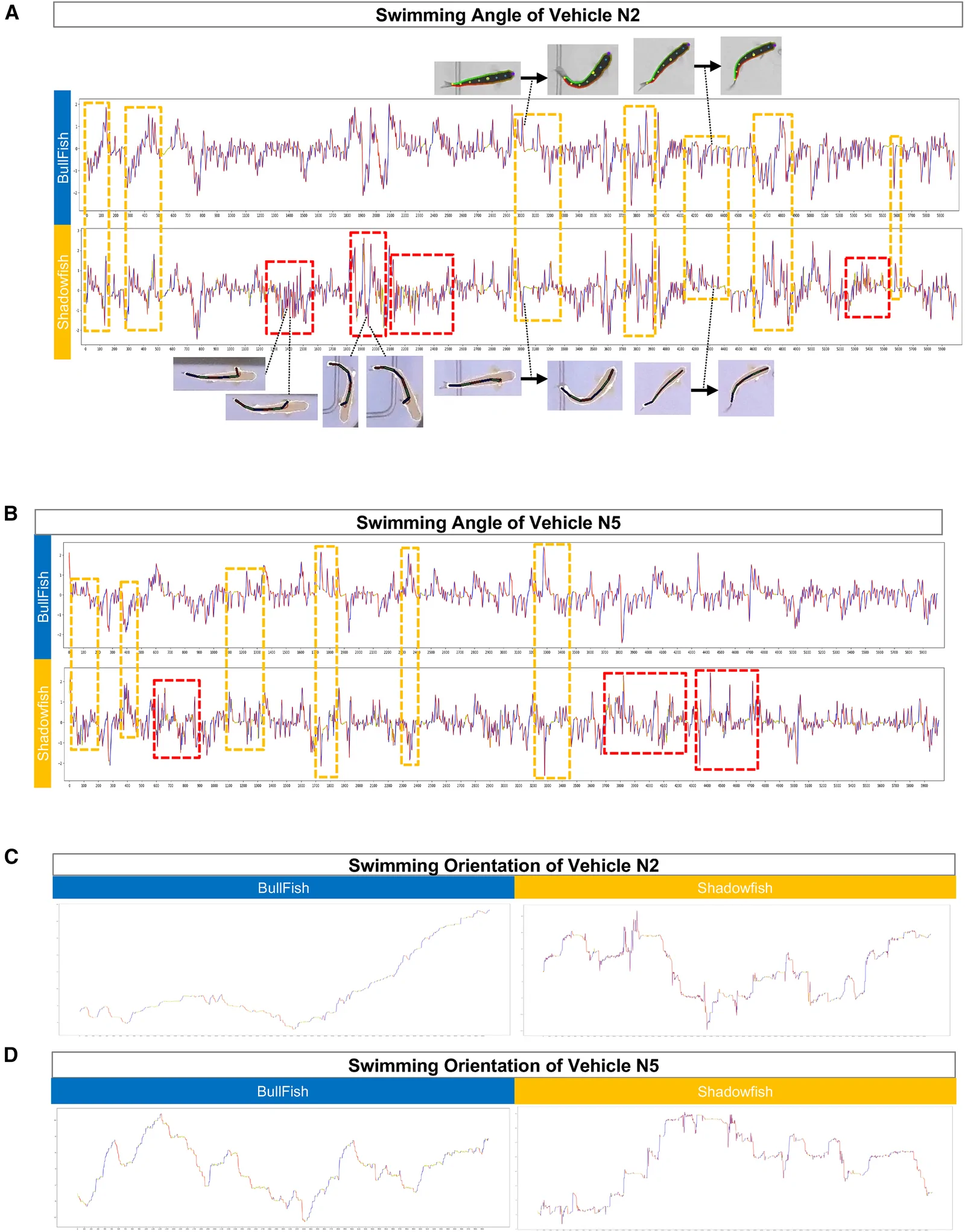
Shadowfish exhibits many more recognition errors in the swimming angle and orientation compared to BullFish (A) Line graph showing the swimming angle of vehicle Zebrafish (N2), where Shadowfish (red) had many more errors compared to BullFish (yellow). Representative figures are shown. (B) Line graph showing the swimming angle of vehicle Zebrafish (N5), where Shadowfish (red) had many more errors compared to BullFish (yellow). (C) Line graph showing the swimming orientation of vehicle N2 analyzed by BullFish (blue) and ShadowFish (yellow). (D) Line graph showing the swimming orientation of vehicle N5 analyzed by BullFish (blue) and Shadowfish (yellow).

While ShadowFish had a reasonably high mean accuracy index of 0.9240, it is insufficient for characterizing individual episodes of zebrafish movement. When bend angle is plotted against time (Figures 7A and 7B), many errors can be spotted from the graph of ShadowFish. Red rectangles show the sections where the midline points were frequently misplaced due to the pectoral fins, leading to noisy spikes that will get misidentified as tail bends. Orange rectangles show the sections where the midline points were reversed, leading to opposite peaks compared to BullFish. The screenshots above the graphs show two selected tail bends within the orange rectangles, first being left (positive) and second being right (negative). BullFish had the correct directions while ShadowFish had the opposite. When orientation is plotted against time (Figures 7C and 7D), the errors of ShadowFish are even more obvious. The whole outline of ShadowFish was completely different from that of BullFish because ShadowFish cannot distinguish between head and tail. There were also sharp spikes along the graph of ShadowFish, which represented midline points reversal or pectoral fin interference.

### Comparative analysis of locomotion parameters reveals differences in values between BullFish and Shadowfish, while no significant differences were found when compared to idTracker

**Figure 8:**
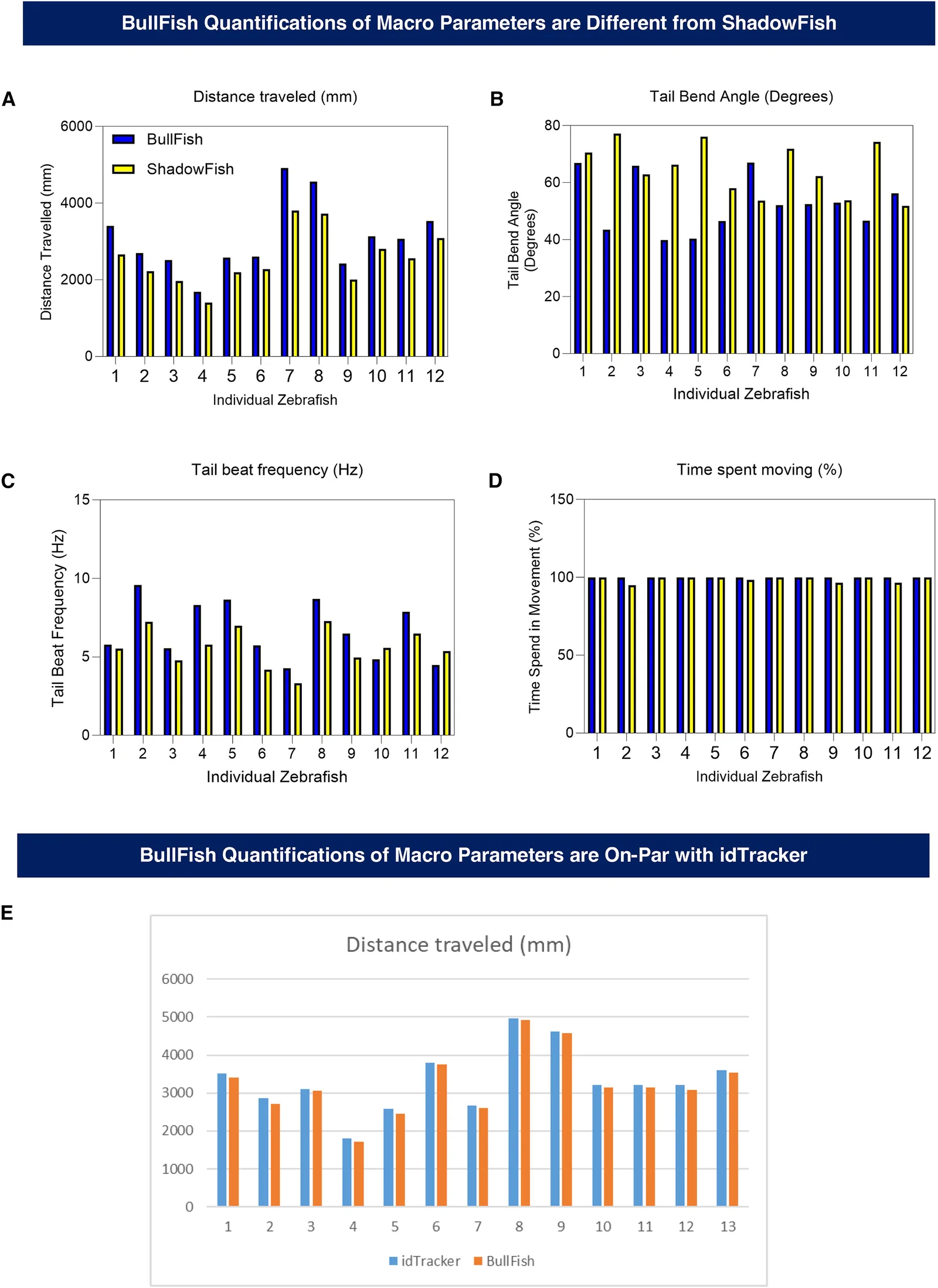
When compared BullFish quantifications of locomotion parameters are more robust than Shadowfish (A) Graph showing that BullFish gives a higher value for distance traveled (mm) compared to Shadowfish. (B) Graph showing that BullFish and Shadowfish produce different values for tail bend angle. (C) Graph showing that BullFish gives higher values for tail beat frequency (Hz) compared to Shadowfish. (D) Graph shows that BullFish and Shadowfish produce similar quantification values for the time spent in movement (%). There are no significant differences between BullFish and Shadowfish. (E) Graph demonstrated that BullFish quantifications of the distance traveled (mm) are on par with idTracker. Data were analyzed for statistical significance using Mann-Whitney U (for two-group comparisons), unless otherwise stated. All data are presented as mean ± SEM, where *p* < 0.05, ∗; *p* < 0.01, ∗∗; *p* < 0.001, ∗∗∗, where 12 individual zebrafish locomotion videos were analyzed.

The comparative analysis of locomotion parameters between BullFish and Shadowfish revealed significant differences in several key metrics. BullFish recorded a higher distance traveled (in mm) compared to Shadowfish, indicating its enhanced sensitivity in quantifying locomotor activity (Figure 8A). This trend continued with tail bend angle measurements, highlighting the variability between the two programs, further emphasizing BullFish robustness in capturing nuanced movement dynamics (Figure 8B). In terms of tail beat frequency, BullFish produced higher values (in Hz) relative to Shadowfish (Figure 8C), suggesting that the former may provide more accurate assessments of fish locomotion, thereby quantifying more events. However, both systems yielded similar results for the percentage of time spent in movement (Figure 8D), where no significant differences were observed between BullFish and Shadowfish. Notably, BullFish quantifications of distance traveled were consistent with those obtained from idTracker (Figure 8E). This alignment underscores the reliability of BullFish in measuring locomotion parameters effectively, at least on par with idTracker.^15^

### BullFish analysis of macro-parameters revealed decreased meandering in the locomotion of 6-OHDA zebrafish

To test the capabilities of the BullFish program in analyzing changes in locomotion, we conducted stereotactic injections of 6-OHDA into the zebrafish brain, specifically targeting the posterior tuberculum (PT).^16^ This region is vital, as it plays a crucial role in integrating sensory and motor functions, influencing behaviors such as swimming and response to environmental stimuli. By administering both 6-OHDA and control saline, we aimed to create a comparative framework that highlights the effects of dopaminergic cell loss on locomotion. The PT’s involvement in processing visual and auditory information further underscores its significance in coordinating movement.^16^ Through this approach, we sought to assess how the BullFish program can effectively capture and analyze the resulting alterations in locomotor patterns, providing insights into the neurobiological mechanisms underlying these changes.

Immunohistochemistry revealed that the 6-OHDA-injected brain contained significantly decreased TH + dopaminergic projections at 3 days post-injection (dpi) (Figures S5A and S5B). Interestingly, microglial activation in the same target region was also significantly increased, indicating a robust inflammatory response to the dopaminergic cell loss. This activation, evidenced by the upregulation of LCP-1+ microglia (Figures S5C and S5D), suggest that the immune response may play a critical role in the pathological processes following neurotoxic insult.

**Figure 9:**
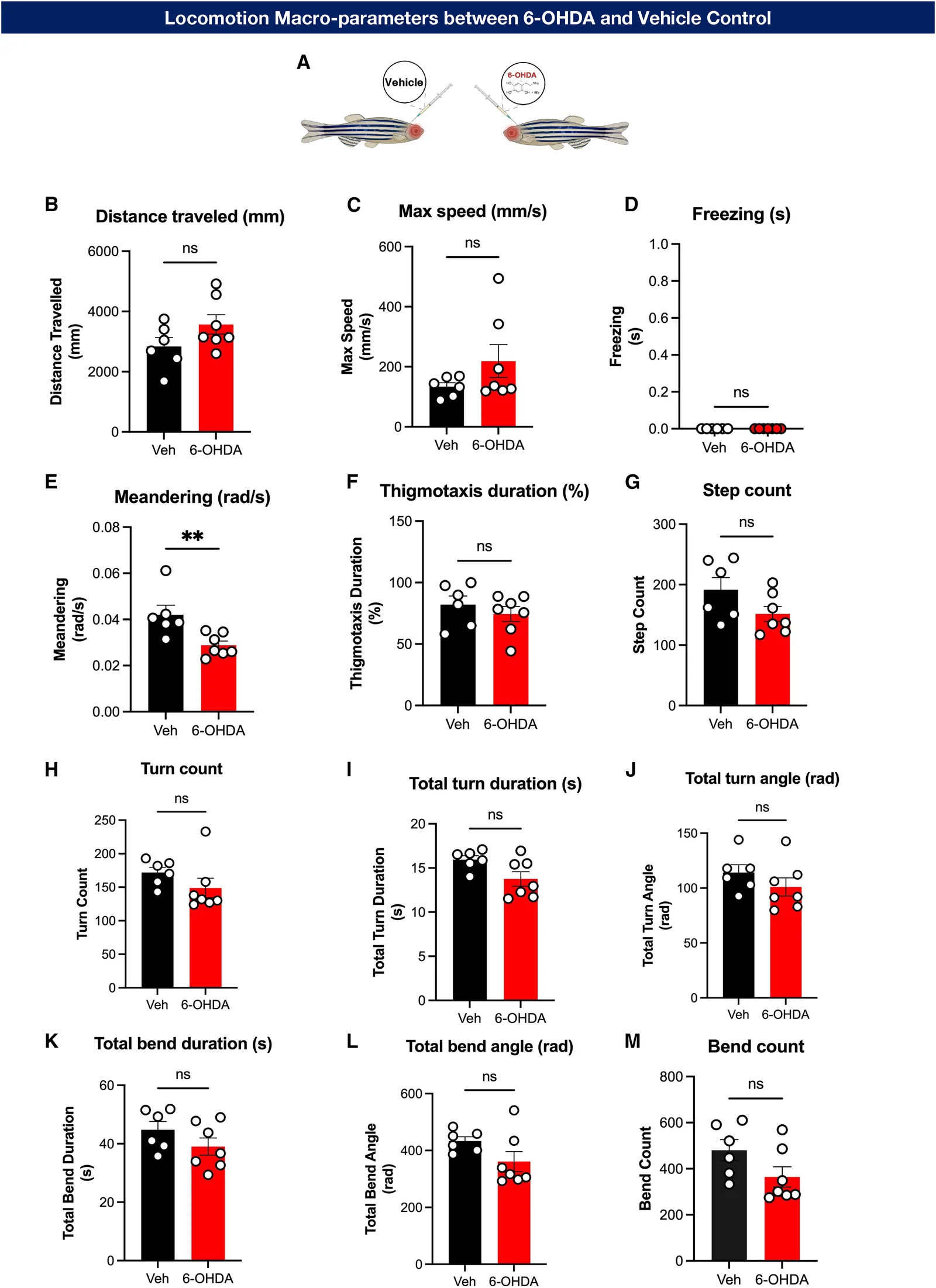
BullFish analysis of macro-parameters revealed decreased meandering in the locomotion of 6-OHDA zebrafish (A) An illustration of the zebrafish brain stereotactic injection of either 6-OHDA or vehicle control. Macro-parameters of zebrafish locomotion were analyzed by BullFish. (B–M) No significant changes between 6-OHDA and vehicle zebrafish were seen in the (B) distance traveled, (C) max speed, (D) freezing, (F) thigmotaxis duration, (G) step count, (H) turn count, (I) total turn duration, (J) total turn angle, (K) total bend duration, (L) total bend angle, nor (M) bend count. However 6-OHDA treatment induced significant decreases in (E) meandering (rad/s) compared to vehicle control zebrafish. Data were analyzed for statistical significance using Mann-Whitney U (for two-group comparisons), unless otherwise stated. All data are presented as mean ± SEM, where *p* < 0.05, ∗; *p* < 0.01, ∗∗; *p* < 0.001, ∗∗∗, where n (6-OHDA) = 7, and n (vehicle) = 6.

This 6-OHDA model is a successful representation of dopaminergic neurodegeneration, as it effectively mimics key cellular changes observed in neurodegenerative diseases. Given these alterations, we are now positioned to examine the motor outcomes in zebrafish, assessing how the loss of dopaminergic function and the accompanying inflammatory response influence locomotor behaviors. BullFish first analyzed a series of videos capturing the locomotion of 6-OHDA treated zebrafish compared to vehicle control. While other macro-parameters showed insignificant change (Figures 9B–9D, 9F, 9G, and 9H–9M). It was revealed that 6-OHDA treated zebrafish have significantly reduced meandering compared to vehicle control (Figure 9E). In this context, diminished meandering may suggest impairments in the basal ganglia circuitry, which is essential for motor planning and execution. Furthermore, reduced meandering could indicate increased rigidity or deficits in adaptive responses to environmental stimuli, mirroring clinical observations in PD.

### BullFish can further stratify locomotion micro-parameters according to speed change, where high speed change results in the most significant behavioral differences after 6-OHDA treatment

BullFish boasts the ability to analyze zebrafish locomotion via several micro-parameters including “bend” and “turn” parameters. This allows for a comprehensive assessment of locomotor behavior, enabling researchers to identify subtle variations in movement patterns and gain deeper insights into the effects of neurodegenerative conditions. However, initial comparisons of micro-parameters in 6-OHDA and vehicle control zebrafish showed no significant changes in several “bend” micro-parameters such as the bend angle traveled, bend position, bend duration, bend wave frequency, bend angular velocity, and bend angle reached (Figures S7A–S7F). Similar non-significance was also observed in the “turn” micro-parameters (Figures S7G–S7I) and “step” micro-parameters (Figures S7J–S7N).

Given these observations, we wondered whether stratification of “bend” and “turn” micro-parameters against speed of swimming could reveal more subtle changes in locomotion as a result of 6-OHDA treatment. By stratifying these parameters against swimming speeds—high, mid, and low—this can reveal specific locomotor alterations linked performance mechanics. Stratifying locomotion analysis based on high, mid, and low speeds is essential due to the differing requirements for agility and muscle use across these ranges. High-speed movement engages fast-twitch fibers for rapid responses, with deficits indicating potential motor control impairments. Mid-speed focuses on speed and maneuverability, highlighting subtle changes that reflect cognitive and motor planning issues. Low-speed swimming involves slow-twitch fibers and cautious movements, where deviations may signify navigation and cognitive challenges. This stratified approach allows researchers to identify specific locomotor deficits linked to neurodegenerative disorders.

**Figure 10:**
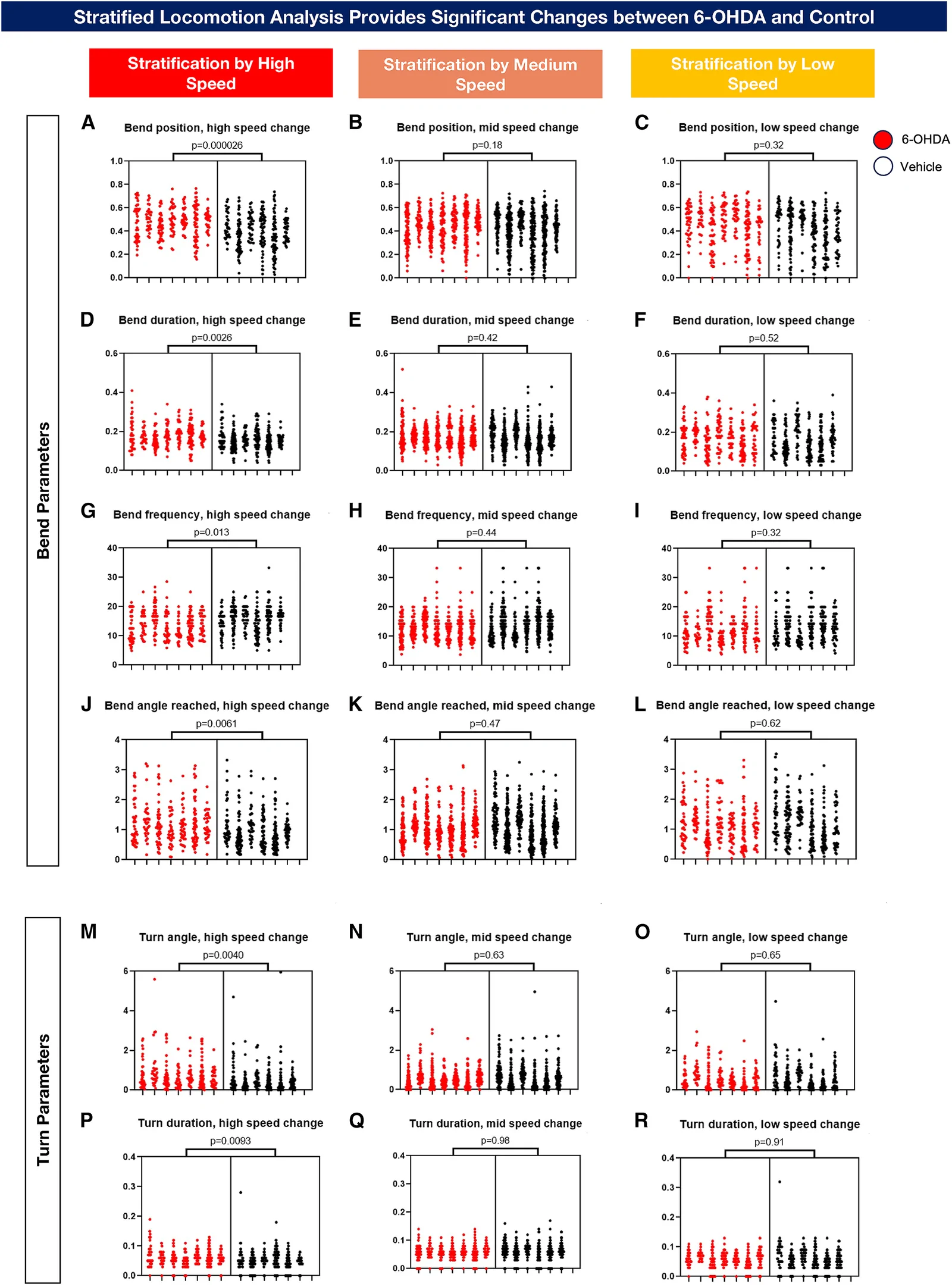
BullFish can further stratify locomotion micro-parameters according to speed change, where high speed change results in the most significant behavioral differences after 6-OHDA treatment (A–R) Bend position is significantly highest in 6-OHDA zebrafish (red dots) compared to vehicle only during (A) high speed change. No statistically significantly changes in bend position were observed in (B) mid speed change or (C) low speed change. Bend duration was significantly higher in 6-OHDA treated zebrafish compared to vehicle during (D) high speed change. No statistically significance changes in bend duration was found in (E) mid speed change nor (F) low speed change. Bend frequency was significantly lower in 6-OHDA injected zebrafish compared to vehicle control only during (G) high speed change. No statistically significant changes were seen in (H) mid speed change nor (I) low speed change. Bend angle was significantly higher in 6-OHDA injected zebrafish compared to vehicle control when stratified against (M) high speed change. No statistically significant changes were observed for (N) mid speed change nor (O) low speed change. Turn duration was significantly increased in 6-OHDA injected zebrafish compared to vehicle control when stratified against (P) high speed change. No statistically significant changes were observed for (Q) mid speed change nor (R) low speed change. Data were analyzed for statistical significance using Mann-Whitney U (for two-group comparisons), unless otherwise stated. All data are presented as mean ± SEM, where *p* < 0.05, ∗; *p* < 0.01, ∗∗; *p* < 0.001, ∗∗∗, where n (6-OHDA) = 7, and n (vehicle) = 6.

To achieve a thorough analysis, BullFish examined changes in micro-parameters by stratifying speed changes into three groups: high, mid, and low for “bend” and “turn” movements. This stratification was based on data spread, categorizing the lowest 25%, mid 50%, and highest 25% of speed changes. Interestingly, bend position was significantly increased in 6-OHDA-injected adults during high-speed changes (Figure 10A), while no significant alterations were noted for mid and low-speed changes (Figures 10B and 10C). Similar trends were observed for bend duration, with significant increases in 6-OHDA zebrafish during high-speed changes (Figure 10D), but no notable differences for mid and low-speed conditions (Figures 10E and 10F). These findings suggest that zebrafish exhibit gait abnormalities that are most pronounced during high-speed swimming, highlighting the importance of dynamic movement analysis in understanding locomotor deficits in neurodegenerative conditions.

Congruent with this hypothesis, we also subjected other swimming parameters to stratify against high-, mid-, and low-speed changes. Indeed, zebrafish injected with 6-OHDA had significantly lower bend wave frequencies than vehicle controls during high-speed change (Figure 10G), which were undetected during mid- and low-speed change (Figures 10H and 10I). Similar trends were observed for bend angle, where 6-OHDA induced changes were significantly found only during high-speed stratification (Figure 10J), but not mid- or low-speed (Figures 10K and 10L). The decreased bend wave frequencies observed in 6-OHDA-injected zebrafish during high-speed changes might reflect the slow alternating movements in patients with bradykinesia due to dopaminergic loss, highlighting the complexity of motor control that basic (unstratified) metrics may overlook.

We proceeded to perform speed-stratified analysis for turn angle and turn duration. In a similar trend, both turn angle and turn duration of 6-OHDA injected was significantly increased compared to vehicle controls during high-speed change (Figures 10M and 10P). No significant changes between 6-OHDA and vehicle controls were seen for turn angle and duration stratified to mid- and low-speed change categories (Figures 10N, 10O, 10Q, and 10R). We report that at our chosen dosage, no significant changes in step length, acceleration, coast percentage, and bend angular velocity were observed between 6-OHDA and vehicle injected zebrafish regardless of speed stratification.

The BullFish protocol highlights the importance of stratifying swimming parameters to uncover subtle locomotor deficits in the 6-OHDA zebrafish model. While traditional metrics did not reveal significant differences following 6-OHDA treatment, our analyses showed that high-speed movements exposed notable abnormalities in bend position, duration, frequency, turn angle, and turn duration. These findings suggest that dopaminergic loss affects motor control primarily during dynamic movements, where the zebrafish exhibited compensatory behaviors that remain undetected at lower speeds. This underscores the necessity of focusing on rapid locomotor responses to fully assess motor dysfunction in neurodegenerative models, offering valuable insights into the complexities of movement disorders akin to those seen in PD.

### The BullFish protocol can investigate gait changes induced by 6-OHDA by studying the asymmetry index (left vs. right) in all macro and micro-parameters

In the preceding sections, we investigated zebrafish locomotion by examining both micro and macro parameters across varying swimming speeds: high, medium, and low. While this analysis sheds light on dysregulated swimming behavior resulting from 6-OHDA-targeted dopaminergic ablation, it is important to recognize that locomotion, and its efficiency, encompasses more than mere velocity. Gait patterns, particularly in PD, serve as critical indicators of movement dysregulation, with gait imbalances often emerging as the first phenotypic manifestation in clinical PD. To address this complexity, the BullFish protocol has been designed to investigate gait changes through a comprehensive analysis of micro and macro parameters. Specifically, we stratify movements based on the left and right sides of the zebrafish body during swimming behavior. By employing an asymmetric index—calculated as *(Right - Left movement parameters)/Total left and right parameters*—we can quantify asymmetries in locomotion. Mathematically, this approach allows us to assess the extent of lateral movement discrepancies, offering insights into gait imbalances that may correlate with clinical phenotypes of PD. Understanding these dynamics is essential for elucidating the underlying mechanisms of gait imbalances in zebrafish locomotion.

**Figure 11:**
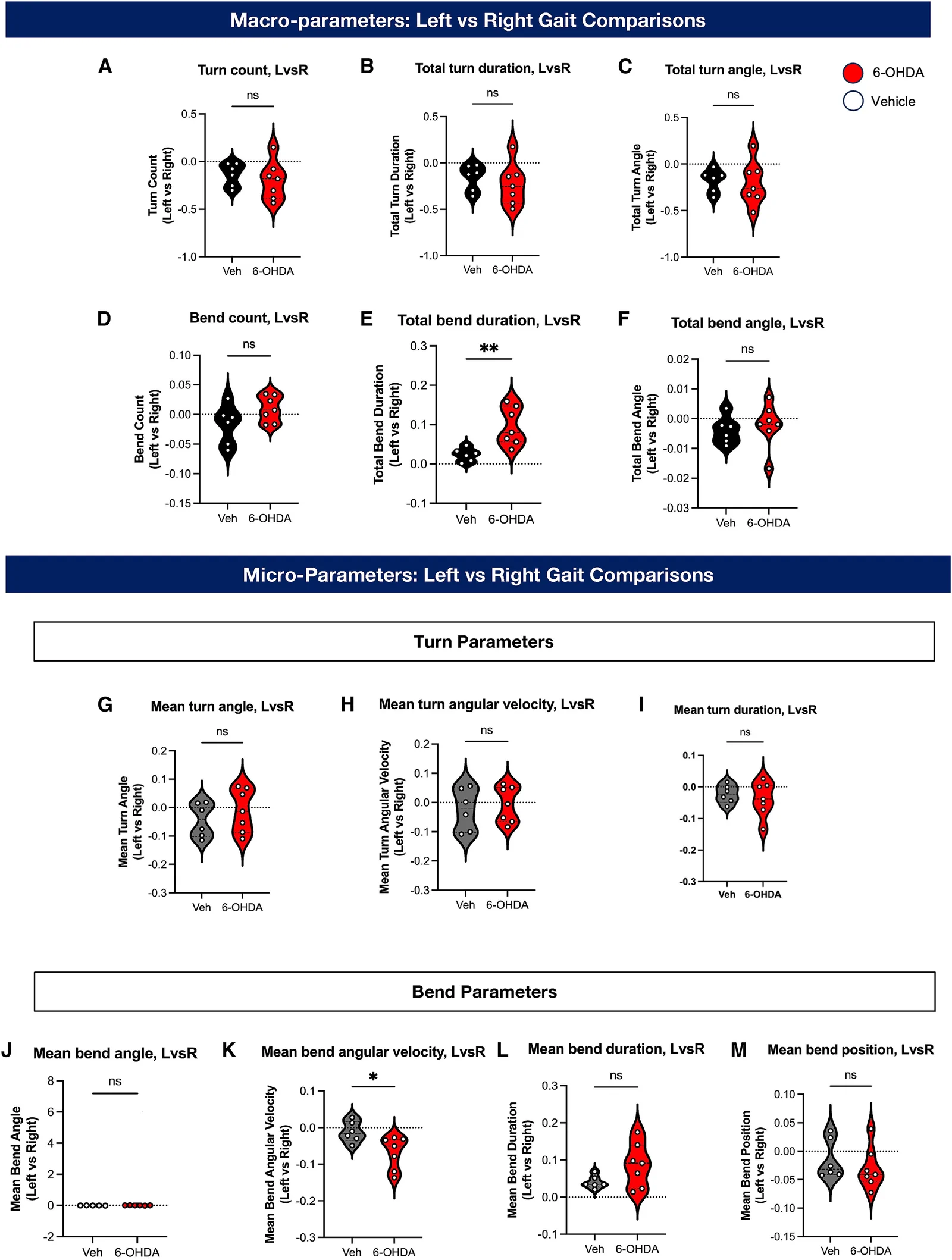
The BullFish protocol can investigate gait changes induced by 6-OHDA by studying the asymmetry index (left vs. right) in all macro and micro-parameters (A–D and F) 6-OHDA treated zebrafish demonstrated no significant changes in the left/right (gait) comparisons of macro-parameters such as (A) turn count, (B) total turn duration, (C) total turn angle, (D) bend count, nor (F) total bend angle. (E, G–J, L, and M) 6-OHDA treatment induced significantly upregulated imbalance of total bend duration (left vs. right body) compared to vehicle control. 6-OHDA treated zebrafish demonstrated no significant changes in the left/right (gait) comparisons of “*turn*” micro-parameters such as (G) mean turn angle, (H) mean turn angular velocity, nor (I) mean turn duration. 6-OHDA treated zebrafish demonstrated no significant changes in the left/right (gait) comparisons of “*bend*” micro-parameters such as (J) mean bend angle, (L) mean bend duration, nor (M) mean bend position. (K) 6-OHDA treatment induced significant reduction of mean bend angular velocity compared to vehicle control. Data were analyzed for statistical significance using Mann-Whitney U (for two-group comparisons), unless otherwise stated. All data are presented as mean ± SEM, where *p* < 0.05, ∗; *p* < 0.01, ∗∗; *p* < 0.001, ∗∗∗, where n (6-OHDA) = 7, and n (vehicle) = 6.

To begin, zebrafish gait was first studied in terms of its macro-parameters. BullFish revealed that 6-OHDA treatment did not result in significant changes in the left/right comparisons across several macro-parameters such as turn count, total turn duration, total turn angle, bend count, total bend duration, nor total bend angle (Figures 11A–11F). We proceeded to perform left/right comparisons of “turn” parameters, and again revealed non-significant effects of 6-OHDA treatment on the mean turn angle, mean turn angular velocity, nor mean turn duration (Figures 11G–11I). Interestingly, left/right comparisons in bend micro-parameters revealed significant decreases of bend angular velocity in 6-OHDA treated zebrafish (Figure 11K). The left/right disparities in bend angular velocity may highlight asymmetrical motor behaviors that mirror dopaminergic imbalances seen in PD. No significant changes were seen in other “bend” micro-parameters such as bend angle, bend duration, nor mean bend position (Figures 11J, 11L, and 11M), but this is attributed to the limitations of 6-OHDA as a drug-induced model, rather than the analytic capabilities of BullFish.

### BullFish stratifies bend micro-parameters to provide enhanced insights into locomotion changes in fin-amputated adult zebrafish

**Figure 12:**
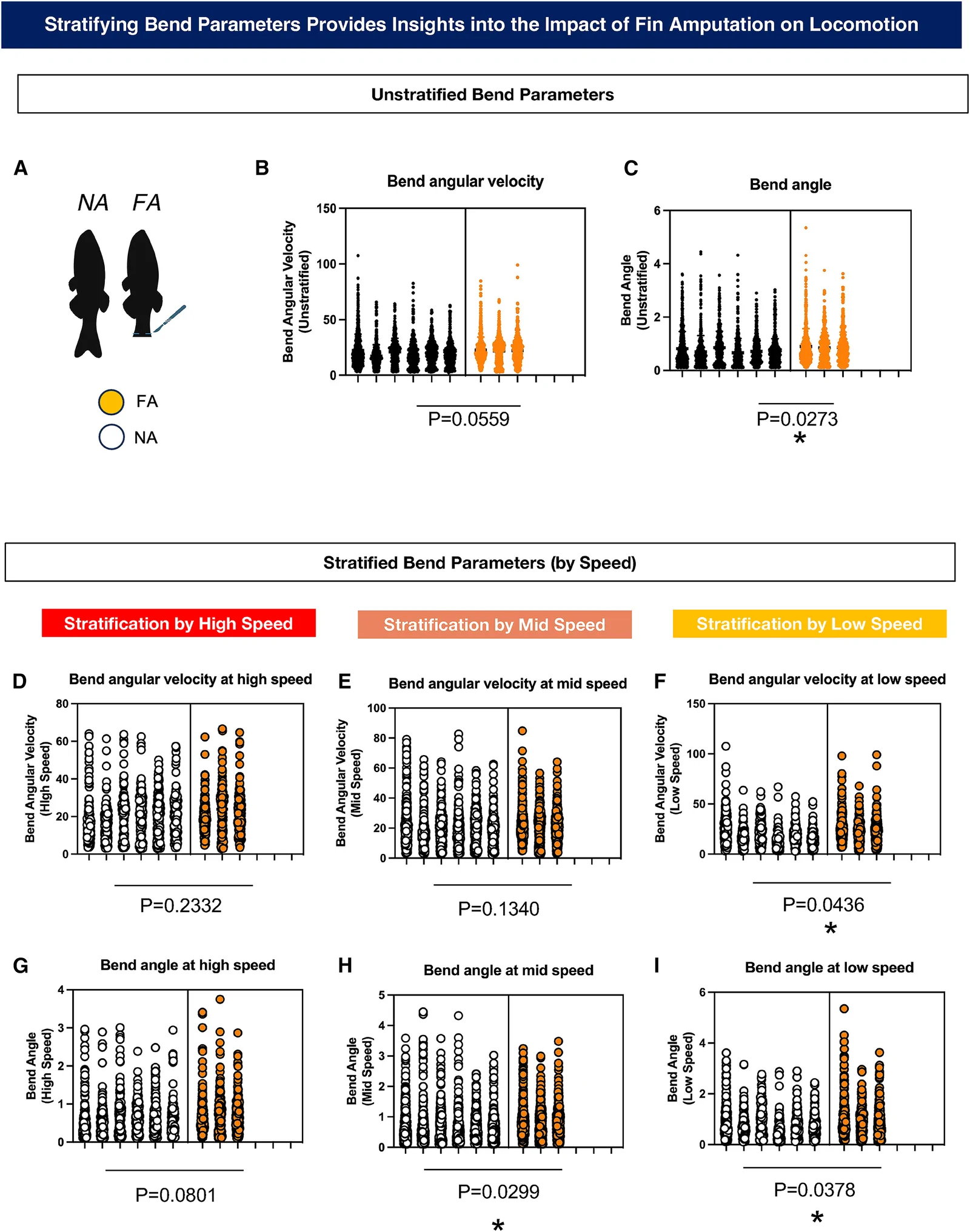
BullFish stratifies bend micro-parameters to provide enhanced insights into locomotion changes in fin-amputated adult zebrafish (A) An illustration of comparisons made between fin amputated (FA) and non-amputated (NA) zebrafish adults. (B) When unstratified, bend angular velocity is not significantly altered between FA and NA zebrafish adults. (C) Even when unstratified, bend angle was significantly upregulated in FA zebrafish compared to NA controls. Bend angular velocity was stratified against (D) high speed, (E) mid speed, and (F) low speed, where FA zebrafish showed significantly increased bend angular velocity compared to NA control. Bend angle was stratified against (G) high speed, (H) mid speed, and (I) low speed, where mid- and low-speed stratifications showed significantly increased bend angles after FA compared to NA controls. Data were analyzed for statistical significance using nested-*t* tests, unless otherwise stated. All data are presented as mean ± SEM, where *p* < 0.05, ∗; *p* < 0.01, ∗∗; *p* < 0.001, ∗∗∗, where n (FA) = 3, and n (NA) = 6.

Next, BullFish was used to explore locomotion in the context of fin amputation in the zebrafish adult. While 6-OHDA represents a pharmacological approach to studying dopaminergic neurodegeneration, fin amputation serves as a model of acute physical injury, allowing for investigations into the immediate biomechanical and behavioral responses of zebrafish. Fin amputation can reveal how physical injuries impact locomotor patterns and recovery mechanisms, particularly in regenerative medicine. This model allows for the assessment of how zebrafish adapt their movement following caudal fin injury, which is essential for understanding the physiological and neurological changes that occur during regeneration (Figures 12A and S6). Furthermore, analyzing locomotion in this context can help elucidate the potential compensatory strategies zebrafish employ post-injury, providing a contrast to the gradual, nuanced progression observed in neurodegenerative models like 6-OHDA.

Upon preliminary observation, BullFish analysis revealed no significant changes in simple macro-parameters after fin amputation (Figures S8B–S8M). This is unsurprising given that macro-parameters such as swimming distance and speed (for example) may only be affected rudimentarily after injury. Furthermore, distal swimming muscles may compensate for the swimming deficiencies cause by a caudal fin amputation. To properly uncover the extent of locomotor disruption after fin amputation, BullFish performed analysis of microparameters.

Although “bend” microparameters such as bend angular velocity was not significantly increased (Figure 12B), stratification by high (Figure 12D), mid (Figure 12E), and low-speeds (Figure 12F), suggested differently. In particular, fin amputation caused the most significant increase in bend angular velocity at low speed stratifications (Figure 12F). This showed that fin amputated zebrafish may rely on increased bending and altered movement patterns during slower swimming, likely as a means to stabilize their locomotion and adapt to the physical loss of fin structure. Various unstratified and stratified bend parameters were found to be insignificant (Figures S10A–S10I), suggesting that these particular locomotion parameters may not be implicated during fin injury.

BullFish also revealed that fin amputation significantly increases the bend angle compared to healthy non-amputated controls (Figure 12C). Further stratification by speed also confirmed significant increases of bend angle particular in mid (Figure 12H) and low speed (Figure 12I) swimming after fin amputation. This significance was however not observed in high speed swimming (Figure 12G). This adjustment in bend angle at mid and low speeds suggests a strategic alteration in swimming mechanics, allowing the fish to navigate effectively despite the injury. Increased bend angle can enhance the surface area engaged in locomotion, improving thrust and control during slower movements. In contrast, the absence of significant changes in bend angle during high-speed swimming indicates that zebrafish might utilize different strategies at higher velocities, possibly relying on faster muscle contractions and streamlined body positions rather than altering the bend angle. This distinction highlights the importance of examining locomotor dynamics across various speed ranges to fully understand the compensatory mechanisms involved in motor adaptation following fin amputation.

### BullFish stratifies turn micro-parameters to provide enhanced insights into locomotion changes in fin-amputated adult zebrafish

**Figure 13:**
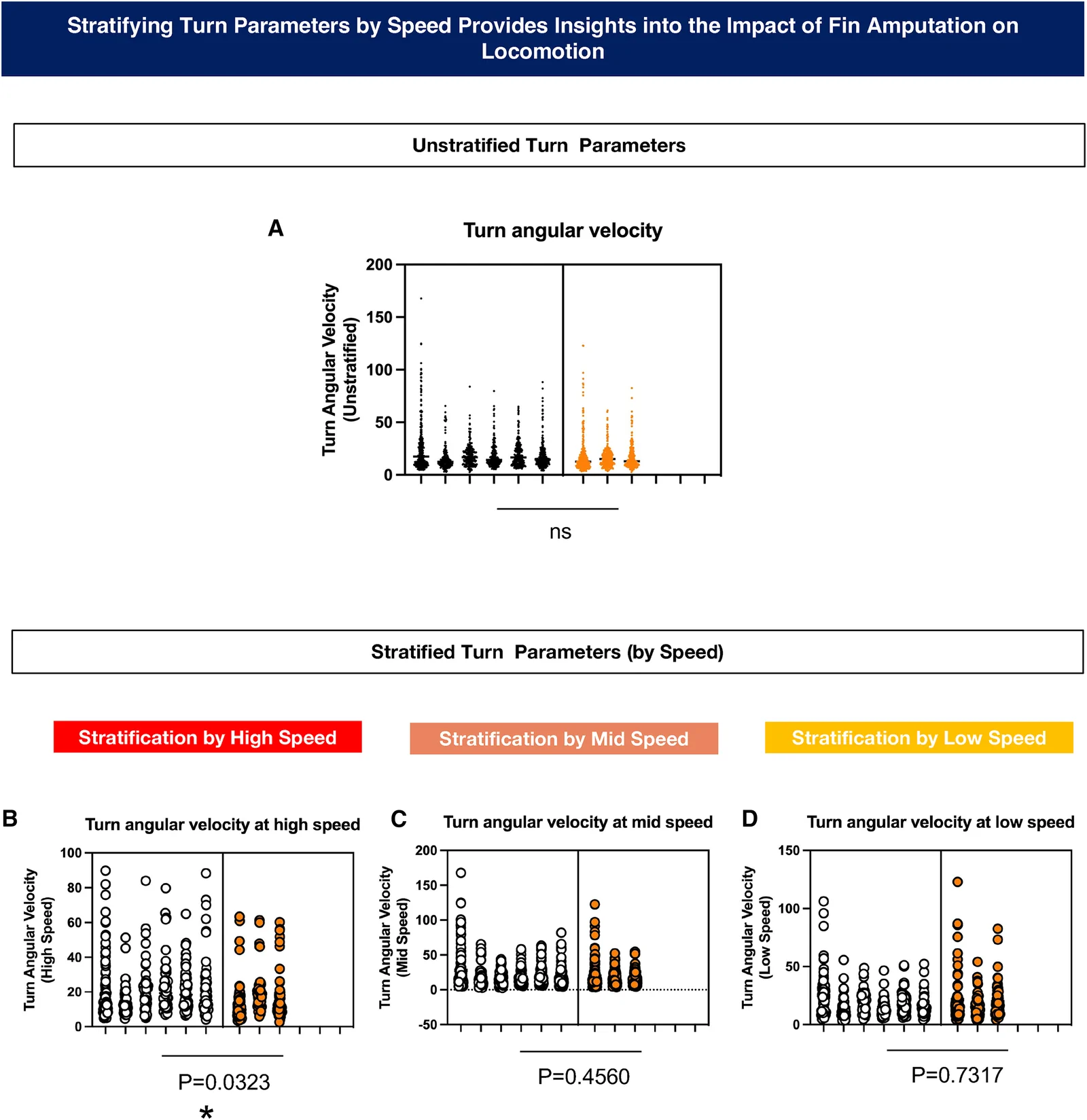
BullFish stratifies turn micro-parameters to provide enhanced insights into locomotion changes in fin-amputated adult zebrafish (A) When unstratified against speed, no significant changes in the turn angular velocity was seen when comparing fin amputated (FA) and non-amputated (NA) control zebrafish. (B) When stratified against speed, turn angular velocity at high speed was significantly decreased in FA compared to NA controls. (C and D) Despite stratification against speed, no significant changes in the turn angular velocity was seen in FA zebrafish compared to NA control during (C) mid, and (D) low speed stratifications. Data were analyzed for statistical significance using Nested-*t* tests, unless otherwise stated. All data are presented as mean ± SEM, where *p* < 0.05, ∗; *p* < 0.01, ∗∗; *p* < 0.001, ∗∗∗, where n (FA) = 3, and n (NA) = 6.

Next, BullFish was utilized to examine the alterations in “turn” micro-parameters following fin amputation. Initial analysis revealed that unstratified “turn angular velocity” did not show significant differences in fin-amputated zebrafish (Figure 13A), indicating that a more detailed stratification is necessary to fully understand the locomotor changes. Stratifying turn angular velocity by speed showed that fin amputation significantly reduces angular velocity specifically during high-speed swimming (Figure 13B) only, but not in mid- and low-swimming speeds (Figures 13C and 13D). To note, BullFish revealed that fin amputation did not significantly alter turn angle nor turn duration before or after stratification by speed (Figures S11A–S11H). This finding indicates that the physical effects of fin amputation may limit the zebrafish’s ability to modify these specific aspects of locomotion, rather than any shortcomings of the BullFish analysis itself. The effectiveness of BullFish lies in its advanced analytical capabilities, allowing for comprehensive assessments of micro-parameters that might not be captured by traditional methods. Its ability to stratify data by various factors provides researchers with deeper insights into the nuances of locomotor adaptations, making it an invaluable tool in the study of locomotion and regeneration.

## Discussion

Zebrafish are increasingly being utilized in neurological research due to their unique genetic and physiological characteristics, making them valuable models for studying various neurodegenerative conditions. As their application in this field grows, it becomes essential to have reliable tools to assess locomotion effectively. Our systematic review of 86 independent studies highlights the significance of zebrafish locomotion analysis across various scientific disciplines, including pharmacology,^17^ neuroscience,^18^ and toxicology.^19^ Despite the increasing demand for robust locomotion metrics, existing platforms like ANY-maze^20^ and Ethovision^21^ often come with substantial financial costs and limitations in the parameters they analyze. In stark contrast, the BullFish protocol not only offers a comprehensive, user-friendly, and free solution for zebrafish locomotion analysis, but also introduces statistics analyzing novel micro-parameters, detailed gait dynamics, and innovative features such as stratification of swimming speeds. By enabling researchers to capture subtle changes in locomotor behavior that traditional methods may overlook, BullFish addresses critical gaps in the current literature and sets a new standard for analyzing locomotion in zebrafish models, particularly in the context of neurodegenerative diseases.

Effective locomotion analysis software must identify and track an animal throughout a video, with advanced systems also characterizing postural changes. BullFish accomplishes both with minimal user input and high accuracy. Most programs only track the position without postural information,^5^^,^^12^^,^^13^ while those that do both often require substantial cost, such as ANY-maze^20^^,^^22^ and Ethovision.^23^ Free programs that achieve both objectives are few, including TRex1,^24^ DeepLabCut,^25^ LACE,^13^ idTracker,^15^ and ShadowFish^12^ are examples.

Although not empirically compared with BullFish in the scope of our study, we briefly explored the capabilities of Trex and DeepLabCut. Both programs leverage deep machine learning for precise posture tracking in animals but require users to undertake the tedious task of labeling numerous images. Given its complicated workflow, BullFish in contrast uses a straightforward algorithm to identify the midline points of zebrafish without any training data, offering a customizable and easy-to-understand solution. BullFish also shares similarities with LACE, as both utilize explicit algorithms to create a pseudoskeleton for analyzing postural changes without requiring training data. A key difference is that BullFish, during binarization, does not perform background subtraction before applying a threshold, which results in the removal of zebrafish pectoral fins. Although pectoral fins play an important role in maneuvering (turning), which are precise but minute movements, they are mostly not involved in burst-and-coast movements,^6^^,^^26^ which is the predominant controlled by the caudal fin in zebrafish locomotion.

Given the similar workflow between BullFish and ShadowFish, we performed numerous side-by-side comparisons through submitting the same set of locomotion videos, and observing the raw data produced. Interestingly, Shadowfish presented several recognition errors, which were correctly recognized in BullFish. For instance, Shadowfish performs errors in fin recognition, and point reversal during extreme turning events during locomotion. Comparisons revealed that while BullFish had no error detection frames, Shadowfish presented on average a 500-fold increase in errors. Pectoral fins actually often interfere with posture recognition, and this problem is severe during our trial runs using ShadowFish.^12^ BullFish is able to remove the fins because the fins are lightly colored, while the body is heavily pigmented. With a light background, the whole body is included in binarization while the fins are left out, thereby resolving the main problems presented in ShadowFish.

BullFish was also directly compared to idTracker,^15^ another open-source software tool designed for tracking and analyzing the movements of individually marked animals in video recordings. We showed that BullFish and idTracker produce almost identical quantification of macro-parameters such as distance traveled during a swimming bout. However, BullFish outperformed idTracker in its ability to analyze micro-parameters and provide detailed insights into nuanced locomotor dynamics, making it a more comprehensive tool for researchers.

While BullFish has a simple and accurate tracking function, the uniqueness of BullFish comes from the concept of analyzing adult zebrafish locomotion with macro- and micro-parameters (definitions of which can be found in Tables S1, S2, and S3). Zebrafish locomotion can be defined by 14 micro-parameters as described. This is similar to gait analysis in humans where stride length, step width, joint angles, etc. are measured.^20^ Generic parameters such as overall speed and freezing are not informative enough because they indicate something is abnormal, but do not tell how the abnormality occurs. Decrease in speed and freezing in 6-OHDA-treated zebrafish have been shown by multiple studies,^17^^,^^27^^,^^28^^,^^29^ but it remains to be verified whether these changes truly represent the parkinsonian phenotype in zebrafish and how the loss of dopaminergic neurons in the zebrafish brain causes these changes. Anxiety is a key example of the confounders,^18^^,^^30^ as it causes freezing and hence decrease in speed. By examining in detail the process of how an organism moves from one place to another, we can not only confirm whether the locomotion pattern fits the disease phenotype but also understand the mechanism of how the disease causes the locomotion phenotype.

Furthermore, locomotion analysis should be further stratified against swimming contexts. Adult zebrafish exhibit a diverse repertoire of locomotion in different contexts, such as exploratory behavior, feeding, and fight-or-flight response. If the parameters are simply aggregated by taking an average and compared, meaningful signals can easily be buried by the wide distribution of data. Stratification allows specific subsets of data to be compared to identify subtle patterns.

To illustrate the significance of this, we replicated a parkinsonian-like zebrafish model induced by widespread TH + dopaminergic loss via 6-OHDA in the PT of the zebrafish brain.^31^^,^^32^ At our chosen dosage of 6-OHDA, there was no significant decrease in distance traveled or average speed or occurrence of freezing episodes, unlike previous studies.^27^^,^^33^ Although slow walking speed and frequent freezing are characteristic features of PD in humans, these manifestations can be very subtle in early disease pathogenesis^34^ and might manifest differently in zebrafish.

To further study minute changes in locomotion, micro-parameters were stratified by the speed change of a step to select those with high-speed change. A high speed change reflects that the zebrafish exerts a large impulse on water, which requires a large bend angle and, or a high bend angular velocity. Selecting steps with high speed change could therefore accentuate abnormalities in parameters related to bending movements. From our results, we can appreciate that to achieve a large speed increase, 6-OHDA zebrafish adopted a more proximal bend position and a larger bend or turn angle, together with increased bend or turn duration and decreased bend wave frequency, in comparison with vehicle controls. Bend and turn angular velocity were not affected. This means that although the speed of bending remained normal, 6-OHDA zebrafish spent more time bending to achieve a larger angle to achieve high speed change. These changes constitute a compensatory response to the slowing of bend wave transmission due to bradykinesia. The generation of thrust depends on the speed of undulatory wave transmission,^11^ and its decrease due to decreased bend wave frequency requires the increase of bend angle and bend angular velocity to maintain the same thrust.

Gait asymmetry between left/right side body movements is a key feature of PD.^9^ Despite its relevance, this has not been analyzed in zebrafish locomotion. BullFish can compare locomotion parameters during high-speed turns to the left and right using an asymmetry index. Interestingly, none of the parameters showed significant asymmetry resulting from 6-OHDA. 6-OHDA zebrafish showed significantly higher degrees of asymmetry of bend angular velocity than vehicle zebrafish, with left smaller than right. This is comparable with bradykinesia causing decreased bend angular velocity. The reason that it did not appear abnormal in previous analysis might be the compensatory increase in right bend angular velocity, illustrating the importance of left-right comparison. Turn angular velocity was however not affected. The difference between turn and bend angular velocity is that turning only describes rotation of the head while bending describes the angle between the head and tail. Tail bend was affected by 6-OHDA while head turn was not affected, and the possible explanation could be bradykinesia causing slowing of the transmission of undulatory wave from head to tail, which causes delayed tail bend and hence decreased bend angular velocity.

Our results indicate that dopaminergic loss leads to marked changes in bend position, bend wave frequency, bend angles, and turn angles, particularly during high-speed movements. Of importance, these parameters have not been addressed within the literature, despite the significance observed in our study. These alterations reflect impaired motor control and mimic the challenges faced by individuals with PD, where patients often exhibit intact motor function during routine activities but struggle with rapid or complex movements.^9^^,^^10^

The implications of these findings extend to understanding the nuanced nature of motor dysfunction in PD. In humans, motor symptoms can vary widely, with many patients demonstrating a phenomenon known as “freezing of gait,” where they experience sudden, brief episodes of an inability to move. This is analogous to the decreased bend frequencies observed in 6-OHDA zebrafish, which may represent impaired motor control. By focusing on high-speed and dynamic movements, our study underscores the necessity of assessing locomotor behavior in a way that mirrors the clinical presentation of PD, where subtle deficits in motor coordination can significantly impact daily activities and overall quality of life.

In contrast, the investigation of fin amputation revealed that macro-parameters remained largely unchanged, reinforcing the notion that traditional metrics might not fully encapsulate the complexities of locomotor adaptations post-injury. This aligns with previous research indicating the compensatory role of distal swimming muscles in fin-amputated zebrafish,^35^ allowing them to maintain activity levels despite structural deficits. Notably, the analysis of bend micro-parameters through stratification by speed showcased that fin-amputated zebrafish rely on increased bending movements during slower swimming to stabilize their locomotion. This finding provides a fresh perspective on the biomechanical and behavioral adjustments made in response to the loss of fin structure. BullFish also uncovered significant increases in bend angle and alterations in turn parameters, particularly at high speeds following fin amputation. The differentiated responses across speed stratifications highlight the need to consider both the type of injury and the speed of movement when assessing locomotor dynamics.

The BullFish protocol stands out in its ability to enhance the analysis of zebrafish locomotion compared to existing methods like ANY-maze and Ethovision. One of the most compelling advantages of BullFish is its cost-effectiveness. While platforms like ANY-maze can involve substantial financial investments, BullFish offers a robust solution for free, making advanced locomotion analysis accessible to a broader range of researchers. Beyond its affordability, BullFish offers a user-friendly interface that simplifies experiment setup and data analysis, allowing researchers to customize parameters for greater flexibility in study design—an essential feature in neurodegenerative research where various factors can influence locomotor behavior. Its comprehensive analytical capabilities distinguish BullFish from other platforms, which often focus solely on basic metrics like swimming speed and distance traveled. BullFish introduces innovative parameters such as bend angles, angular velocities, and stratified swimming speeds, enabling the detection of subtle changes in locomotion that may indicate early signs of neurodegeneration. Additionally, the protocol facilitates detailed quantification of gait dynamics, addressing a critical gap in zebrafish locomotion studies and providing valuable insights into how dopaminergic loss affects movement. This level of detail is crucial for researchers aiming to develop targeted therapies for conditions such as PD.

The BullFish protocol surpasses traditional metrics that focus predominantly on swimming speed by introducing a comprehensive suite of parameters, including bend angles, angular velocities, and stratified swimming speeds. Specifically, it implements high-speed stratifications to capture subtle changes in locomotor behavior following treatments like 6-OHDA, allowing for a more nuanced understanding of motor deficits. Additionally, BullFish analyzes left/right discrepancies during high-speed swimming, providing insight into potential asymmetries in locomotion. This stratified approach is crucial for detecting nuanced variations that mirror the rapid movement challenges faced by patients with conditions like PD. By enhancing current understanding of zebrafish locomotion through these detailed analyses, BullFish equips researchers with critical tools to evaluate motor function more accurately, facilitating the identification of biomarkers for early diagnosis and the development of targeted therapeutic strategies.

### Limitations of the study

Primarily, our analysis if zebrafish locomotion was conducted in a two-dimensional (2D) space, which inherently limits the robustness of the locomotion assessment. Three-dimensional (3D) analysis^17^^,^^23^^,^^27^ would allow for a more comprehensive evaluation of zebrafish locomotion, capturing vertical movements and providing a fuller picture of their behavioral dynamics. 3D analysis is particularly relevant because zebrafish exhibit various behaviors along the *z* axis, such as diving and hovering, which are critical for understanding their locomotor strategies in different environments. The absence of real-time analysis further constrains the ability to observe immediate changes in locomotion in response to various stimuli, potentially missing key insights into dynamic motor behaviors.

Furthermore, it is important to acknowledge certain limitations inherent in using the 6-OHDA zebrafish model for PD research. While the 6-OHDA effectively induces dopaminergic cell loss, it does not capture the full spectrum of phenotypes associated with PD. PD presents not only with motor symptoms but also with cognitive (PDD),^36^ emotional, and autonomic dysfunctions,^37^ which are not replicated in a simplified drug-induced PD zebrafish model. Thus, while the model provides insights into specific aspects of motor dysfunction, it should be viewed as a piece of the larger puzzle in understanding the multifaceted nature of PD.

In conclusion, the BullFish protocol offers a significant advancement in zebrafish locomotion analysis, enabling detailed examination of gait dynamics and subtle motor deficits associated with neurodegenerative conditions. Its ability to stratify swimming speeds and assess left/right asymmetries provides valuable insights that current methods often overlook. By making advanced analysis more accessible, BullFish enhances our understanding of movement disorders and may facilitate the identification of targeted therapeutic interventions.

## Resource availability

### Lead contact

Requests for further information and resources should be directed to co-corresponding author, Sherry Sin-Hang Yeung (sherrysh@hku.hk).

### Materials availability

This study did not general new hardware.

### Data and code availability

• All data reported in this paper will be shared by the lead contact upon request.
• All original code has been deposited at https://github.com/CH2BrCH2N3/BullFish.
• Full BullFish Guide can be found at https://connecthkuhk-my.sharepoint.com/:w:/g/personal/u3579172_connect_hku_hk/IQABprvCmEAdTZAbOXo6eW5MAadQy0LzFCHN_epVYhN-EZo?e=jEv5UB.

## Acknowledgments

We would like to express our gratitude to all the researchers and institutions that contributed to the foundational work in zebrafish locomotion analysis, which has significantly informed this study. In particular, we would like to extend our gratitude to *Ho-Hin Chan*, *Zita Man-Wai Ng*, *Elvina Man-Yui Ho*, *Jenny Jia-Ying Xu* for their work during the summer of 2023. We also thank *Angelina Hei-Yee L*i for drafting and checking references (bibliography) used within this study. The authors thank colleagues in the Center for PanorOmic Sciences (CPOS, HKU) and the Center of Comparative Medicine Research (CCMR, HKU). Finally, we thank the General Research Fund (17121622) from the Research Grant Council of the Hong Kong SAR Government, and Seed Funding for Basic Research (202011159177, 202111159215, and 2309100700) to R.C.-C.C. and G.T.-C.W. L.N.D. is also supported by Bingei and L&T Charitable Foundation Professorship in Dementia Research to R.C.C.C.

## Author contributions

S.S.-H.Y. and H.-M.C. contributed equally to the study. S.S.-H.Y. conceptualized this study. H.-M.C. performed programming and softcode preparations. Zebrafish behavior experiments were performed in contribution by W.-H.C., C.-K.L., H.-M.C., and S.S.-H.Y. Quantifications for the systemic review were performed by S.S.-H.Y., C.-K.L., and Y.-Q.L. These were verified by H.-M.C. Figures were made in joint efforts by S.S.-H.Y. and H.-M.C. Manuscript was written by S.S.-H.Y. and H.-M.C., and reviewed by G.T.-C.W. and R.C.-C.C. The BullFish userguide was written by H.-M.C. We thank the funding acquired by G.T.-C.W. and R.C.-C.C. which greatly supported the development.

## Declaration of interests

The authors declare no competing interests.

## STAR★Methods

### Key resources table

**Table:** 

REAGENT or RESOURCE	SOURCE	IDENTIFIER
**Antibodies**		

Lcp1 antibody	GeneTex	Cat#GTX124420
Tyrosine Hydroxylase Polyclonal Antibody	Invitrogen	Cat#PA5-85167

**Chemicals, peptides, and recombinant proteins**		

6-OHDA	Sigma Aldrich	Cat#162957

**Experimental models: Organisms/strains**		

AB Zebrafish	ZIRC	RRID:ZIRC_ZL1
Tg(mpeg1:egfp)	ZIRC	RRID:ZIRC_ZL9940

**Software and algorithms**		

BullFish_Tracker	This publication	https://github.com/CH2BrCH2N3/BullFish
BullFish Guide	This publication	https://connecthkuhk-my.sharepoint.com/:w:/g/personal/u3579172_connect_hku_hk/IQABprvCmEAdTZAbOXo6eW5MAadQy0LzFCHN_epVYhN-EZo?e=jEv5UB
Shadowfish	Hughes, G.L. et al.*, 2020*	Reference [^12^]
idTracker	Pérez-Escudero, A et al.,2014	Reference [^15^]

### Experimental model and study participant details

All experiments were conducted according to rules and regulations outlined by the Committee on the Use of Live Animals in Teaching and Research (CULATR) in the University of Hong Kong, under CULATR#5697-21. Zebrafish wildtype strains AB and Tg(mpeg1:egfp) were obtained from the Zebrafish International Resource Centre (ZIRC), and randomized as both genders (sex) were used in this study. Zebrafish used in this study ranged from ages 7 days-post-fertilization (dpf) to 13 months-post-fertilization (mpf). These animals were maintained using a 12:12 hour light-dark cycle at 28°C. All wild types were spawned through in-house crossing. Zebrafish larvae were maintained at 0.0002% methylene blue solution (Sigma-Aldrich, M9140) made in E3 medium for the first 24 hours post-fertilization (hpf). They were bleached according to standard protocols. Juveniles (>3 months post-fertilization, mpf) were subsequently raised in system water and fed three times daily – twice with 1:1 Otohime/Sparos feed, and once with *Artemia s.*

### Method details

#### Eligibility criteria for systematic review

Eligibility criteria for the meta-analysis were determined via inclusion and exclusion criteria listed below. To summarize, exclusion criteria for the meta-analysis include (i) non-experimental studies (i.e. reviews, abstracts, commentaries, meta-analyses, commercial products, non-peer reviewed appraisals); (ii) protocols involving alternative species to the zebrafish adult, (iii) studies involving zebrafish embryos, larvae or juveniles (21dpf), (iv) locomotion paradigms involving small wells, and finally (v) studies not originally published in English. To capture recent advancements in the field, this systemic review chose to only involve studies published between January 2013 to July 2023.

#### Search strategies for systematic review

Studies included in this systematic review were isolated through two databases (i) PubMed (NIH) and (ii) Google Scholar®. On the 15th of September 2023, the following complete search terms were submitted through PubMed (NIH): *“(Zebrafish Adult Locomotion) NOT (review)) NOT (larvae)) NOT (embryos)) NOT (mice)) NOT (mouse)) NOT (meta))”.* On the 20th of September 2023, the following search terms were submitted through Google Scholar®: *“Adult Zebrafish Locomotion*”. These searches were further filtered for papers published between January 2013 to July 2023, and manually again according to our inclusion and exclusion criteria. Searches from both databases were cross-checked individually to remove duplications Out of a total of 232 search results, 86 appropriate studies were finally isolated for full systematic review.

#### Stereotactic injection of 6-OHDA into the posterior tuberculum of the zebrafish brain

In zebrafish adults, 99.96mM of 6-OHDA (Sigma Aldrich, 162957) was injected into the posterior tuberculum (PT) of the anesthetized zebrafish through a custom-made stereotactic frame. To describe the injection protocol briefly, 6-OHDA was prepared as described by Vijayanathan et al. (2021). The site of the right PT was identified using AZBA brain atlas. Following successful localization, a burr hole was introduced into the skull bone above the PT site using a portable micro-aluminium handheld drill (Yakamoz), fitted with a 27-gauge bevelled surgical needle. Using the Nikon SMZ 800N stereomicroscope (Nikon®, SMZ800N), the glass micro-capillary was adjusted for the AP-axis according to *y-value*, ML-axis according to *x-value*, and finally lowered on the DV-axis according to *z-value* to allow injection of 50 nL of 6-OHDA into the right PT. Zebrafish were subjected to behavioural locomotion testing, or sacrificed for pathological staining of TH and LCP-1 at 3 days-post-injection (dpi).

#### Locomotion behaviour paradigm

Zebrafish were evaluated on locomotor abnormalities at 3 dpi after 6-OHDA or 9 dpa of Fin Amputation. Recording took place within 1200-1600 to reduce swimming discrepancies. It was conducted in a custom-made chamber consisting of a hole on the top for camera placement, and an additional lower compartment for white backlight installation, which was an iPad showing white screen. The zebrafish exploration tank consisted of a custom-made plastic rectangular swimming tank measuring 210 mm x 144 mm x 140 mm filled to approximately 50 mm in depth with system water. The tank was translucent to blur zebrafish reflections while allowing adequate illumination. To begin the behavior paradigm, zebrafish adults were introduced into the exploration tank and allowed to habituate for 5 minutes to remove novelty stressors that may influence locomotion. Following that, free-roaming swimming behavior was recorded for 5 minutes using a 100-fps camera (Insta360 ONE RS, Arashi Vision Inc.). To note, system water in the exploration tank was replaced for each zebrafish. Blueprints for the behavior chamber can be found in **the BullFish USERGUIDE**.

#### The bullfish protocol

The locomotion videos were analyzed using the BullFish program we developed. It was developed in Python 3.11.12 using Spyder 5.5.1 as the Integrated Development Environment (IDE) and Miniconda 25.1 for managing Python packages. OpenCV 4.9 is the key Python library used for video handling and zebrafish tracking. Detailed instructions of how to use BullFish can be found in **the BullFish USERGUIDE**.

The following focuses on how statistical analysis is performed by BullFish to compare 6-OHDA and vehicle injected zebrafish. After obtaining the coordinates representing the zebrafish center and midline in each frame of a video, BullFish identifies individual episodes of undulation (steps) of the zebrafish and characterizes them by 13 parameters as described in Tables S1 and S2. Hence, the data structure of our locomotion experiment consists of two groups (6-OHDA and vehicle), 13 zebrafish (7 for 6-OHDA and 6 for vehicle), and 2343 steps (Each zebrafish had 117 to 266 steps). To allow comparison of the steps between 6-OHDA and vehicle injected zebrafish, a mixed effects model was used for each of the 13 parameters. For example, to compare the speed change in a step between 6-OHDA and vehicle, the speed changes of all steps of all 6-OHDA injected zebrafish are compared with those vehicle injected. The code for statistical analysis can be found at BullFish/mixedlm.py at main · CH2BrCH2N3/BullFish. It reads the output data of BullFish and outputs a csv file containing the p value for each comparison. The mixed effects models were computed using the statsmodels 0.14.4 library in Python. Afterwards, the steps were stratified by each parameter into three strata, low, medium, and high, and compared correspondingly. For example, the steps can be stratified into three strata with low, medium, and high-speed change. The stratification was done independently in each zebrafish, with cutoffs being determined by the first and third quartiles of the stratifying parameter for that zebrafish. For example, to compare the turn angle at high-speed change between 6-OHDA and vehicle, the steps of each zebrafish are stratified by speed change and then selected such that the steps have the top 25% speed change of that zebrafish. A mixed effects model was similarly used to test for difference in turn angles between 6-OHDA and vehicle. The code can be found at the same link.

#### Cryosections and immunohistochemistry of the zebrafish whole brain

Zebrafish adult brains were dissected and subjected to fixation overnight with 4% PFA at 4°C. Next, whole zebrafish brains were subjected to submersion of 30% sucrose for two consecutive days at 4°C. To obtain cryosections, the zebrafish whole brain was embedded on optimum cutting temperature compound (OCT) and sectioned coronally to be 10 microns thick. Slides were stored in -80°C until needed for immunohistochemistry. To begin immunostaining, slides were thawed at room temperature and subjected to three 5-minute washes. Slides were subjected to an incubation of 10% normal goat serum made in 0.3%-Triton PBS, and probed for primary antibody (**TH**, 1:250) overnight at 4°C. After this, slides were washed three times for 5-minutes and counterstained with Alexa Fluor secondary antibody. Finally, slides were probed with 1xDAPI and mounted with DAKO fluorescent mounting medium. Confocal images were obtained through Carl Zeiss LSM900 confocal microscope, using a 20x air lens unless otherwise described. Imaging was performed on 7 micron thick sections, which have been imaged with confocal at a layer interval of 0.25 micron (approximately 28 slices, stacked together).

### Quantification and statistical analysis

Quantifications for the systematic review were performed using Microsoft Excel (Microsoft) and plotted on GraphPad PRISM10 (Dotmatics). The Accuracy Index (AI), can be calculated by the number of error frames detected by BullFish_analysis divided by the total number of frames of the analyzed video segment. Confocal micrographs were transformed from z-stacks to orthogonal projections using ZEN Blue® (Carl Zeiss). Confocal micrographs were quantified by mean fluorescent intensity (MFI) using Fiji/ImageJ (NIH). Behavioural statistics were analyzed using the BullFish protocol and plotted on PRISM10. For statistical analysis, normality tests were performed to determine the distribution of data points. Unpaired independent t-test were used for normalized data, while Mann-Whitney U test was used for non-normalized data points between wildtype and 6OHDA injected zebrafish. All data are presented as mean ± S.E.M. Statistical significance on graphs were demonstrated as as p ≤ 0.0001∗∗∗∗, p ≤ 0.001∗∗∗, p ≤ 0.01∗∗, p ≤ 0.05∗, p>0.05 (non-significant).

## Supplemental information

Document S1. Figures S1–S11

Table S1. The four quantities represent zebrafish position and posture

Table S2. List of macro-parameters in BullFish

Table S3. List of micro-parameters in BullFish
