# Supplementary material for: BullFish: Software for an automated stepwise analysis of positional and postural kinematics of zebrafish locomotion
Source: iScience. 2026 Apr 17;29(6):115798. doi: 10.1016/j.isci.2026.115798 (PMC13156763; doi:10.1016/j.isci.2026.115798)
Supplement: Document S1. Figures S1–S11 [file mmc1.pdf]

iScience, Volume *29*

Supplemental information

BullFish: Software for an automated stepwise analysis of positional and postural kinematics of zebrafish locomotion

Sherry Sin-Hang Yeung, Ho-Ming Cheng, Chak-Kwan Lee, Wai-Hang Chan, Yong-qi Luo, Gordon Tin-Chun Wong, and Raymond Chuen-Chung Chang

Supplementary Figure 1

A

Databases (Query)	Analyzed	Without Locomotion	Alternative Species	Larvae/ Embryo	Non-Experimental	Well Apparatus	Non-English Studies	Duplicated Studies
PubMed(NIH) Google Scholar®	**86**	159	3	6	11	1	1	6

B

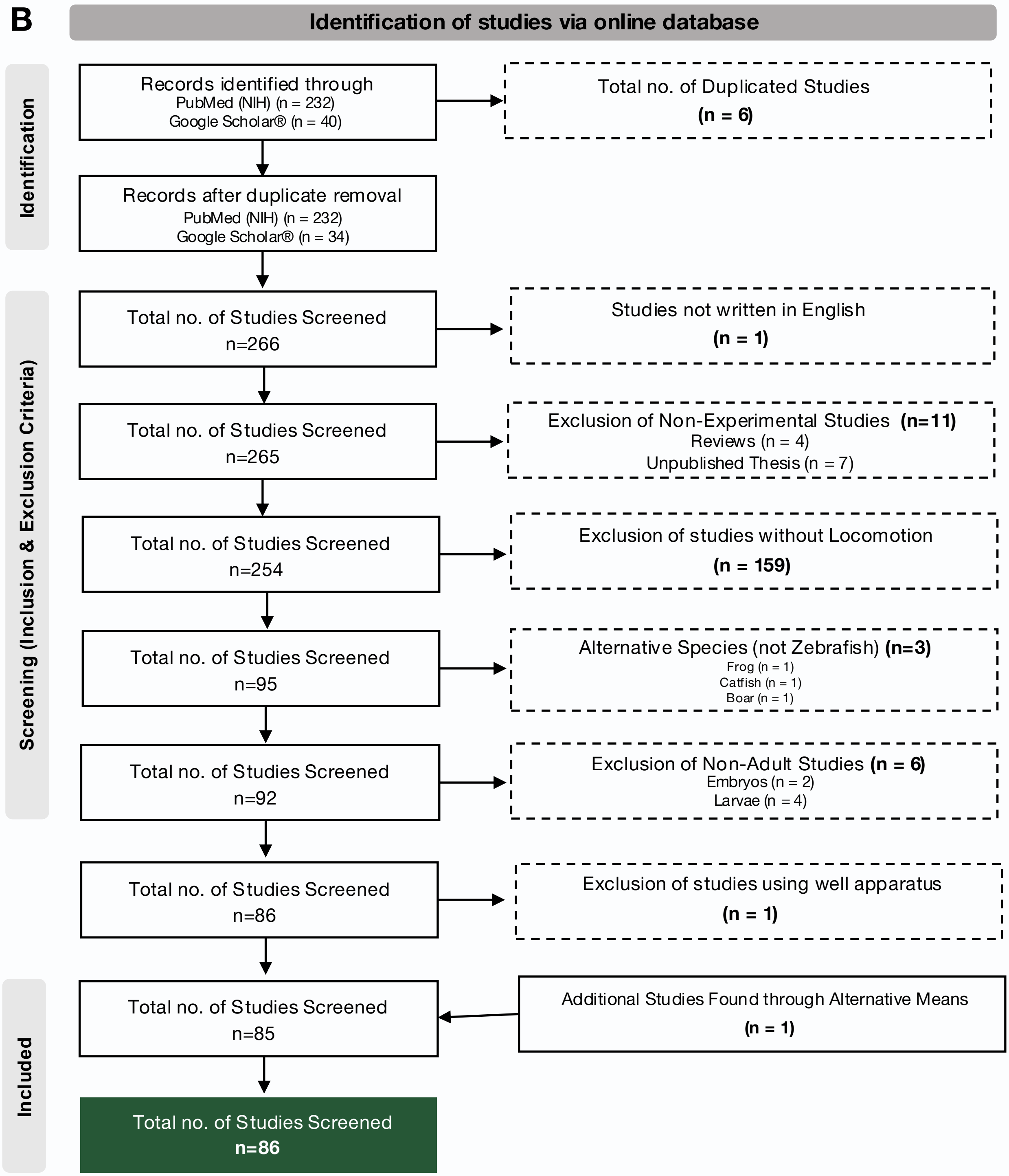

Supplementary Figure 1. Papers included in this systematic review was determined using search results generated from database via PRISMA protocols. (A) In short, 272 studies were collectively isolated from PubMed (NIH) and Google Scholar® searches. After removing duplicates and accounting for inclusion/exclusion criteria, 86 studies were independently analyzed for this meta-analysis. **(B)** The Preferred Reporting Items for Systematic Reviews and Meta-Analysis (PRISMA) was followed to generate a flow chart outlining identification, screening (inclusion/exclusion criteria) and inclusion process of relevant studies in the meta-analysis.

Supplementary Figure 2

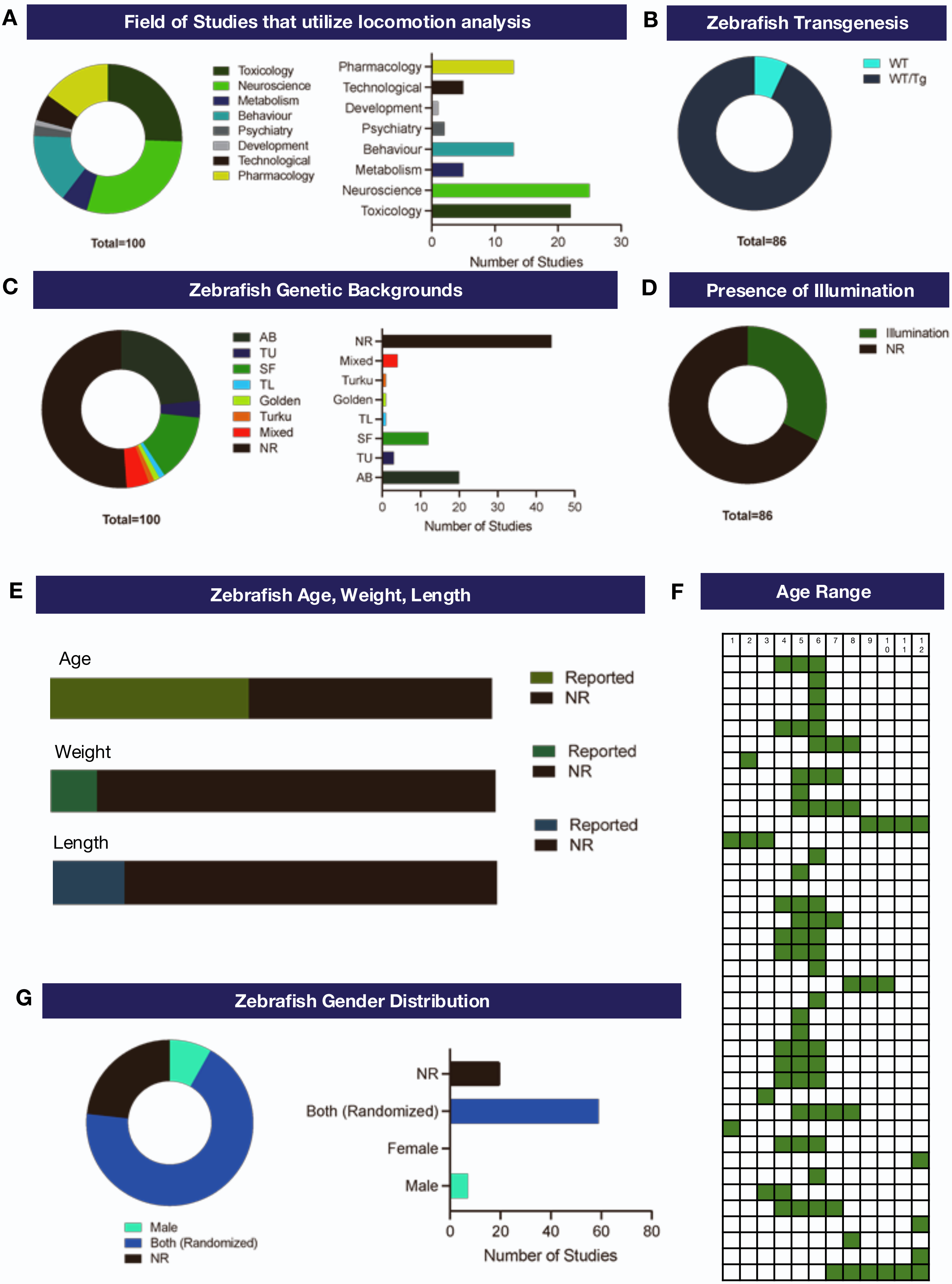

Supplementary Figure 2. Locomotion analysis was extensively applied in a wide range of *Danio rerio* (zebrafish) studies irrespective of strain, transgenesis, genetic background or age. (A) Pie-chart (left) demonstrating that zebrafish locomotion was found to be utilized in fields such as toxicology (25.6%), neuroscience (29.1%), metabolism (5.8%), behaviour (15.1%), psychiatry (2.3%), development (1.2%), biotechnology (5.8%), and pharmacology (15.1%). Bar chart (right) showed the number of studies that utilized locomotion analysis. **(B)** Pie chart demonstrating that approximately 93% of studies involved locomotion analysis of both wildtype and transgenic zebrafish, while 7% only examined wildtype locomotion behaviour. **(C)** Zebrafish locomotion was analyzed in a variety of backgrounds. Where AB (23.3%), TU (3.49%), SF (14%), TL (1.2%), Golden (1.2%), Turku (1.2%), Mixed (4.7%) were reported. Meanwhile approximately 51.2% of studies did not report (NR) the background of Danio rerio. **(D)** Approximately 32.6% of studies reported the use of illumination during locomotion analysis, while 67.4% did not. **(E)** Age of zebrafish were reported in approximately 45.4 pf the studies (NR, 54.7%). Weight of the zebrafish were reported in only 10.5% of the studies (NR, 89.5%). Length of the zebrafish was reported in 16.28% of the studies (NR, 83.7%). **(F)** Majority of zebrafish locomotion was analyzed in fish ranging from 4-6 months post fertilization. **(G)** Zebrafish locomotion was analyzed in randomization of both genders (68.6%), male (8.1%), female (0%), where NR= 23.3%. Quantifications were performed as %, unless otherwise indicated.

Supplementary Figure 3

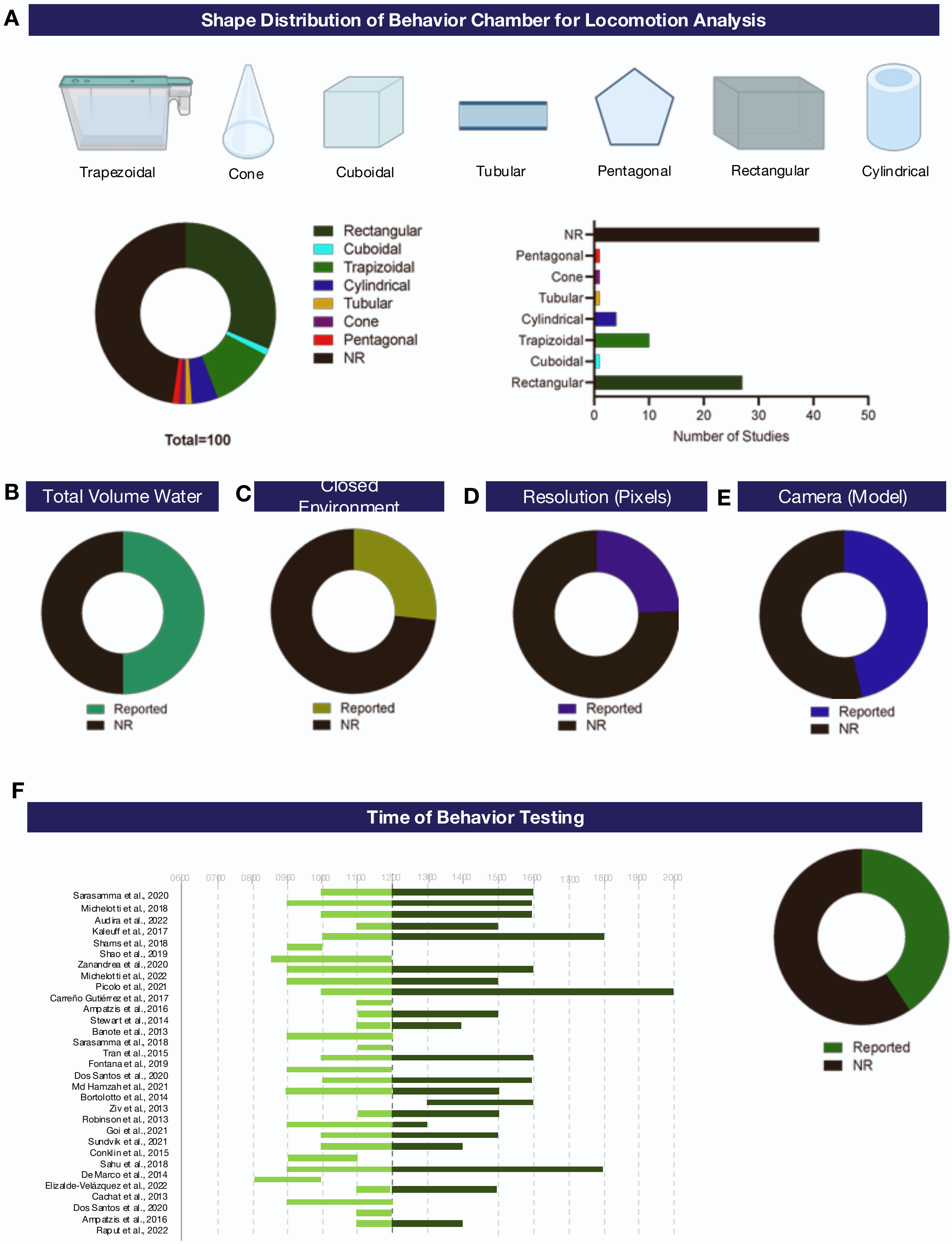

Supplementary Figure 3. Various hardware considerations are important for the analysis of locomotion in *Danio rerio* (zebrafish). **(A)** Studies report the use of behaviour chambers of different shapes. Pie chart illustrating the use of rectangular (31.4%), cuboidal (1.2%), trapezoidal (11.6%), cylindrical (4.7%), tubular (1.2%), cone (1.2%), pentagonal (1.2%) shapes. **(B)** The total volume of water was reported in approximately 50% of the studies (NR, 50%). **(C)** Approximately 26.7% of studies reported the use of a closed environment. **(D)** Approximately, 24.4% of studies reported the resolution of the acquired locomotion data (NR, 75.58%). **(E)** Approximately, 40.7% of studies reported the model of the used camera for data acquisition (NR, 59.3%). **(F)** A progression bar chart demonstrating times when locomotion experiments were performed. Approximately 67% of studies reported when the locomotion paradigm was performed.

Supplementary Figure 4

A Zebrafish locomotion can be analyzed through a variety of software available

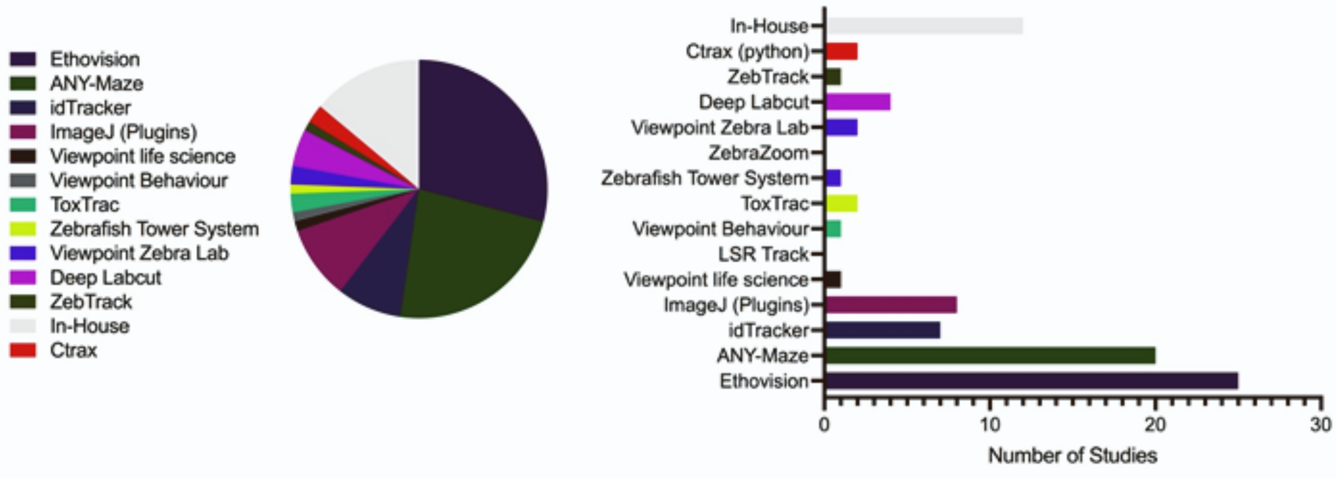

B Frame rate of Locomotion Recording

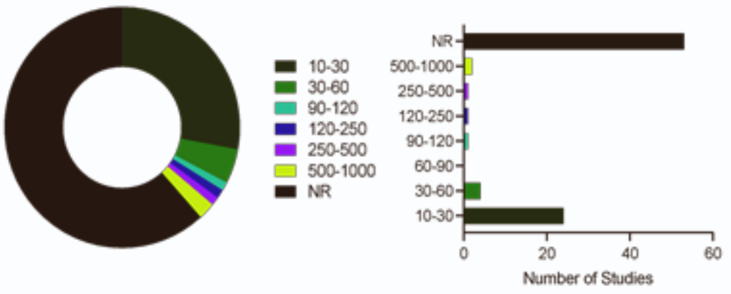

C 2D or 3D Locomotion Analysis

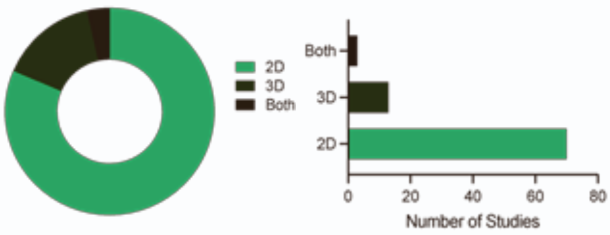

D Quantified Locomotion Parameters

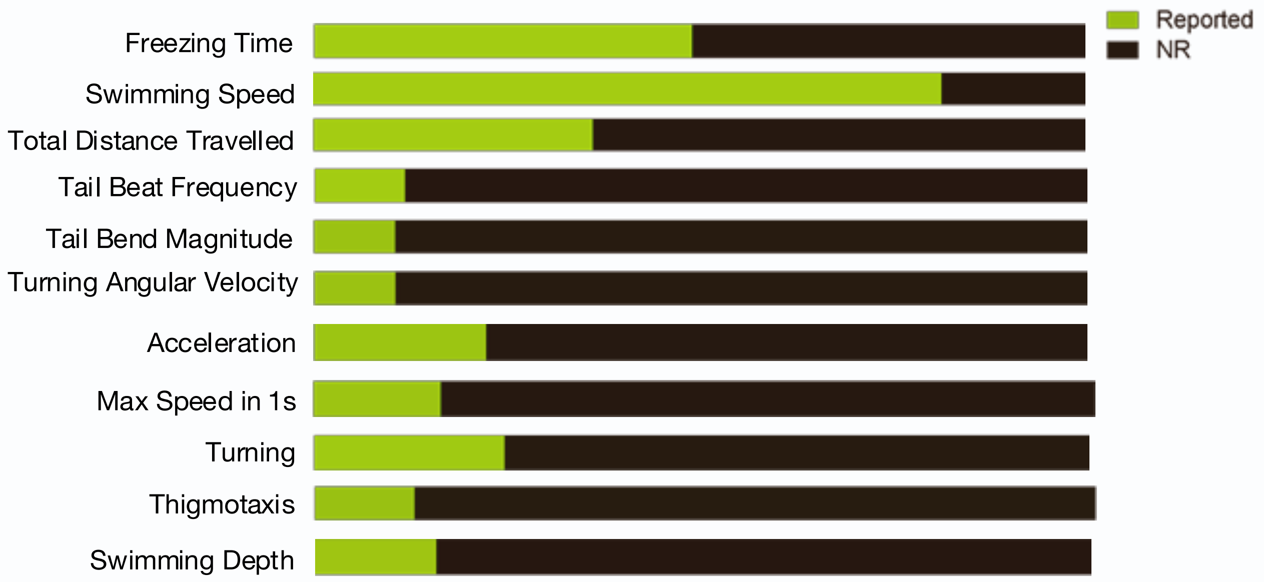

Supplementary Figure 4. Locomotion of *Danio rerio* (zebrafish) were analyzed through various programs, methods and parameters. **(A)** A pie chart demonstrating the preferable use of several software for the analysis of zebrafish locomotion. **(B)** Pie chart [left] and bar charts [right] demonstrating that the Frame rate was mostly not reported in most zebrafish locomotion studies. Most studies utilized a framerate of 10-30 fps. **(C)** Pie chart demonstrating that locomotion was mostly performed in 2-dimensional view, rather than 3-dimentional. **(D)** Chart demonstrating the most popular parameters quantified in zebrafish locomotion analysis.

Supplementary Figure 5

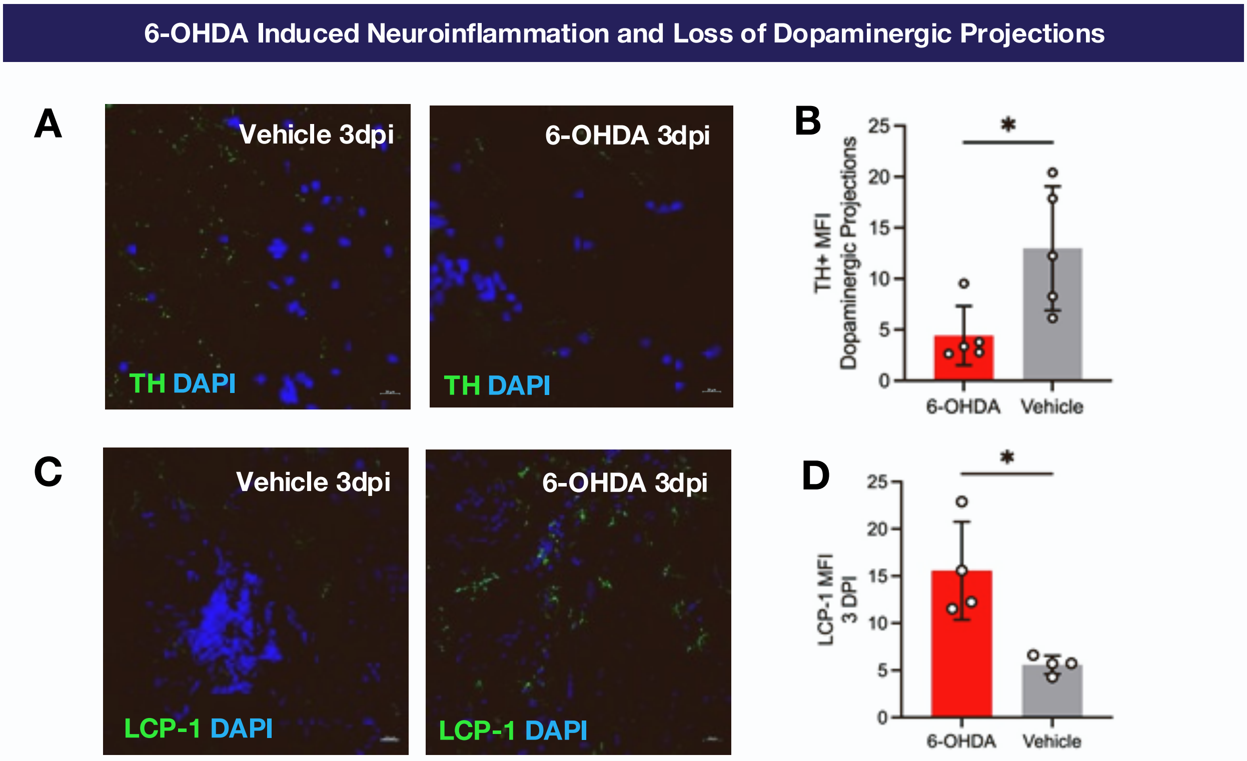

Supplementary Figure 5. 6-OHDA injections induced significant loss of dopaminergic projections at 3 days-post-injection in the right posterior tuberculum compared to vehicle control. (A) Confocal images of the posterior tuberculum of 6-OHDA injected zebrafish demonstrate significantly lower TH+ (green) dopaminergic projections. **(B)** Quantifications of TH+ mean fluorescent intensity (MFI) was significantly lower in 6-OHDA (red bar) compared to vehicle zebrafish. **(C)** Confocal images of the posterior tuberculum of 6-OHDA injected zebrafish demonstrate higher lcp-1+ (green) microglia compared to vehicle control. **(D)** Quantifications of lcp-1+ mean fluorescent intensity (MFI) was significantly higher microglia activation in 6-OHDA zebrafish (red bar) compared to vehicle control. IHC was performed on 7 micron thick sections, which have been imaged with confocal at a layer interval of 0.25 micron (approximately 28 slices, stacked together). Scale bar is approximately 20 microns. Data was analyzed for statistical significance using Mann-Whitney U (for two-group comparisons), unless otherwise stated. All data are presented as mean ± SEM, where $p<0.05$, *; $p < 0.01$, **; $p < 0.001$, ***, where n=5.

Supplementary Figure 6

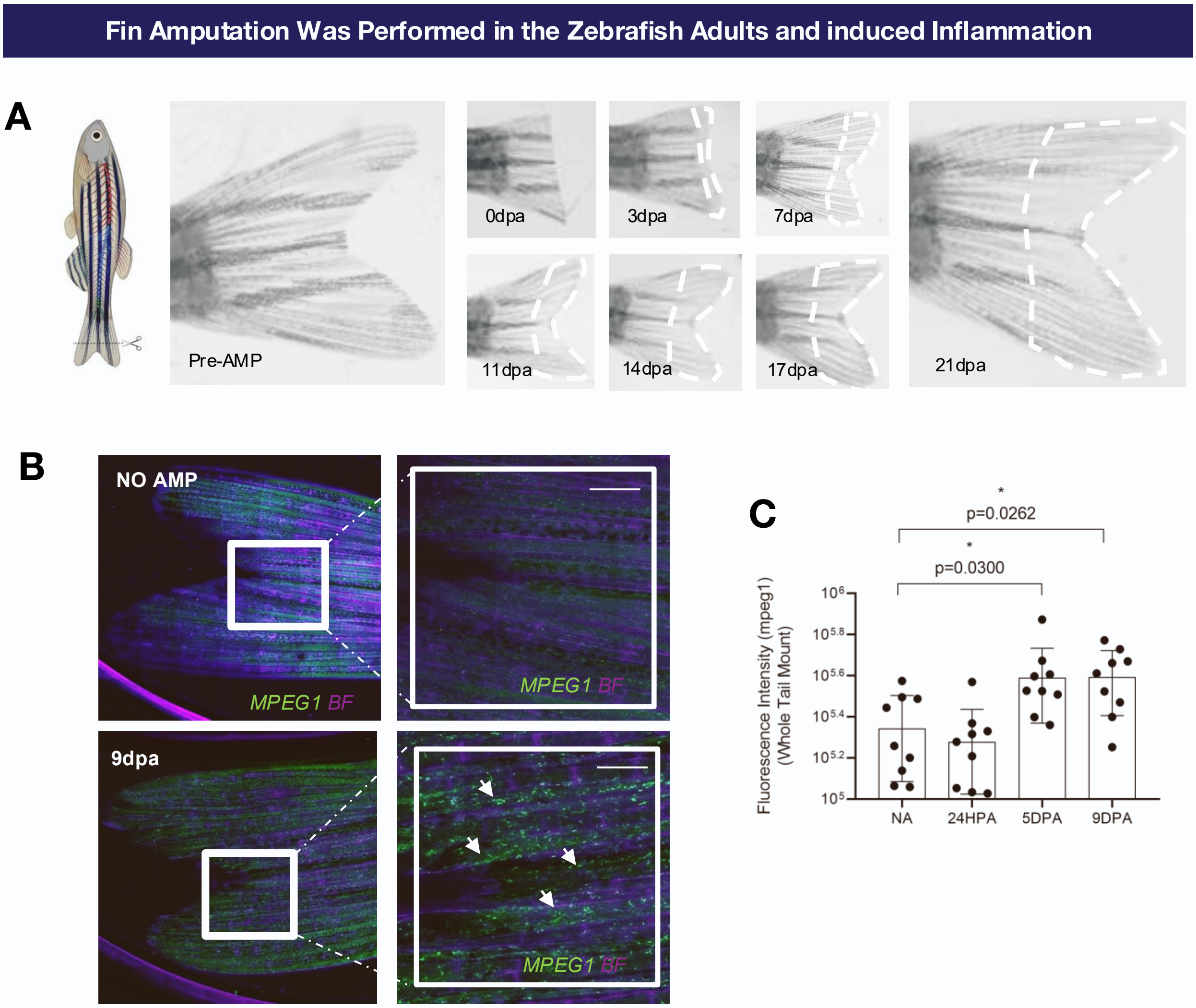

Supplementary Figure 6. Fin Amputation was performed in zebrafish and induced significant inflammation via the recruitment of mpeg1^{+} macrophages. (A) Brightfield imaging was used to capture physiological changes to the caudal zebrafish fin from pre-amputation, 0 days-post-amputation (dpa), 3, 7, 11, 14, 17, and 21 dpa. Typically, 21 dpa is when the fin regenerates completely. **(B)** Confocal microscopy reveals increased numbers of mpeg1^{+} macrophages (green) in the caudal fin at around 9 dpa compared to non-amputated (NA) control. **(C)** Quantifications of the mean fluorescent intensity of mpeg1+ in the zebrafish tail reveals significant upregulation at 5 and 9 dpa. The latter was chosen to examine the effects of sustained inflammation. Scale bar is approximately 100 microns. Data was analyzed for statistical significance using One-way ANOVA, unless otherwise stated. All data are presented as mean ± SEM, where $p<0.05$, *; $p < 0.01$, **; $p < 0.001$, ***, where n=9.

Supplementary Figure 7

Unstratified Locomotion Analysis Provides No Significant Changes Between 6-OHDA and Control

Unstratified Bend Parameters

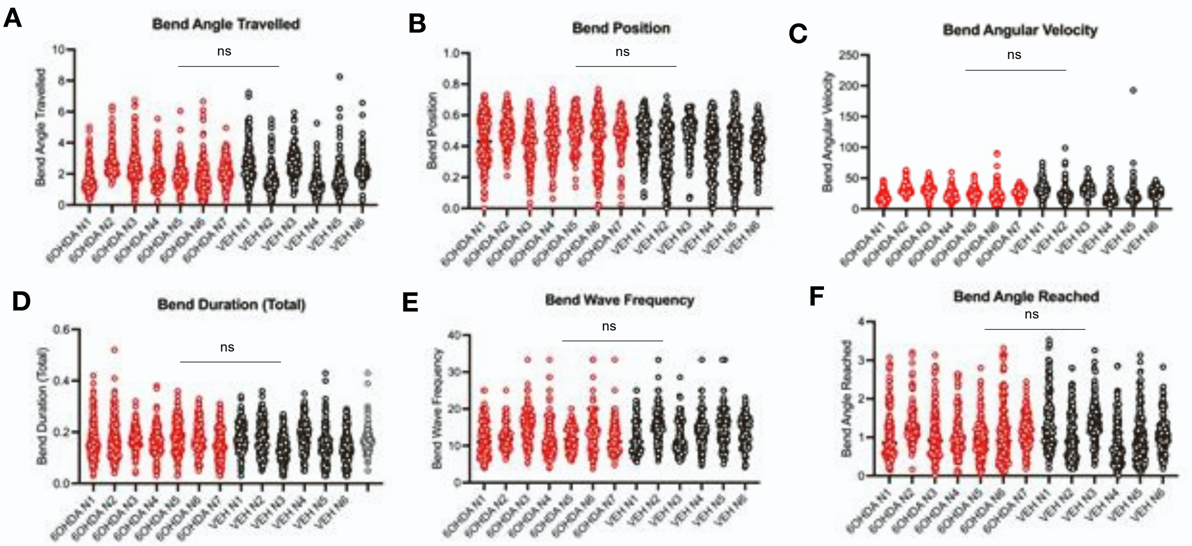

Unstratified Turn Parameters

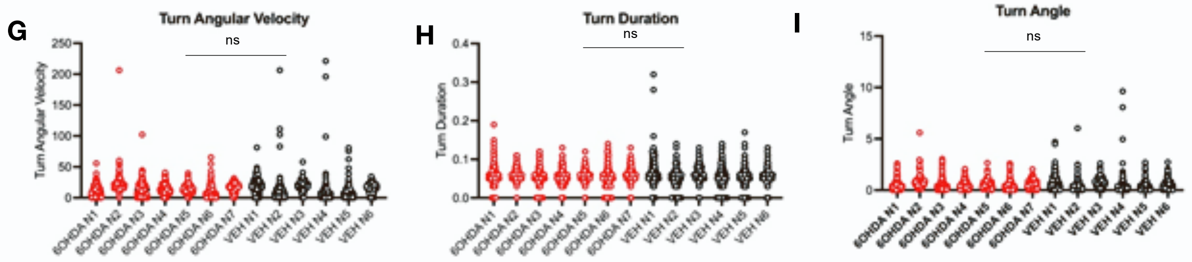

Unstratified Step Parameters

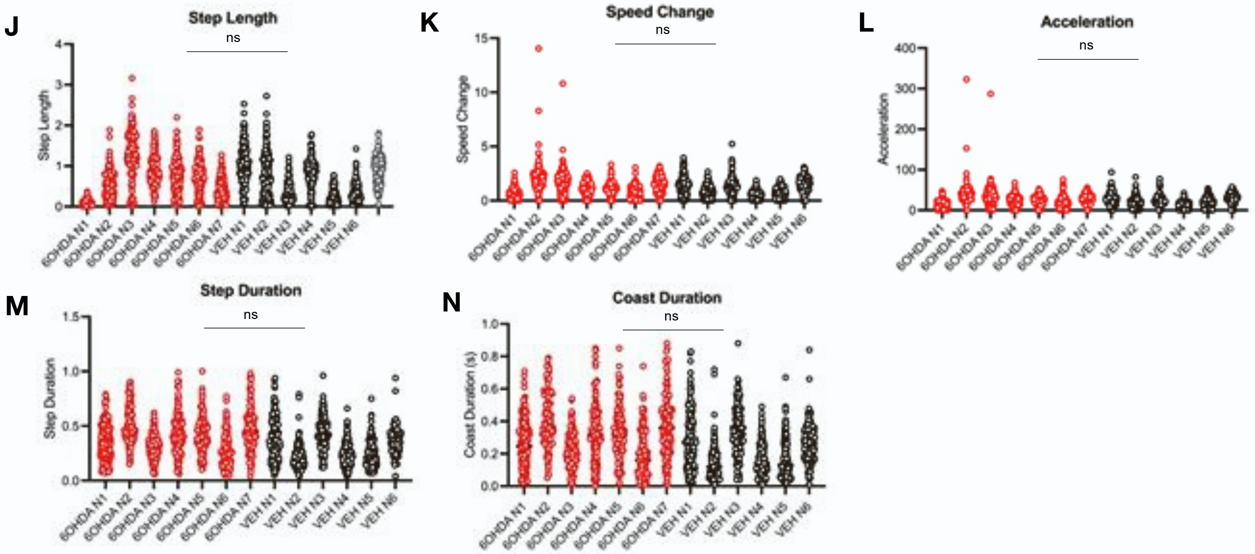

Supplementary Figure 7. Unstratified Locomotion Analysis of ‘Bend’, ‘Turn’, and ‘Step’ Parameters Revealed No Significant Changes Between 6-OHDA and Control. No significant changes between 6-OHDA (red) and vehicle controls (black) were observed in unstratified ‘bend’ parameters such as **(A)** bend angle travelled, **(B)** bend position, **(C)** bend angular velocity, **(D)** bend duration (total), **(E)** bend wave frequency, (F) bend angle reached. No significant changes between 6-OHDA and vehicle controls were observed in unstratified ‘turn’ parameters such as **(G)** turn angular velocity, **(H)** turn duration, nor **(I)** turn angle. No significant changes between 6-OHDA and vehicle controls were observed in unstratified ‘step’ parameters such as (J) step length, **(K)** speed change, **(L)** Acceleration, **(M)** Step Duration, nor **(N)** Coast Duration. Data was analyzed for statistical significance using One-way ANOVA, unless otherwise stated. All data are presented as mean ± SEM, where no significance was shown (6OHDA) = 7, and n (vehicle) = 6.

Supplementary Figure 8

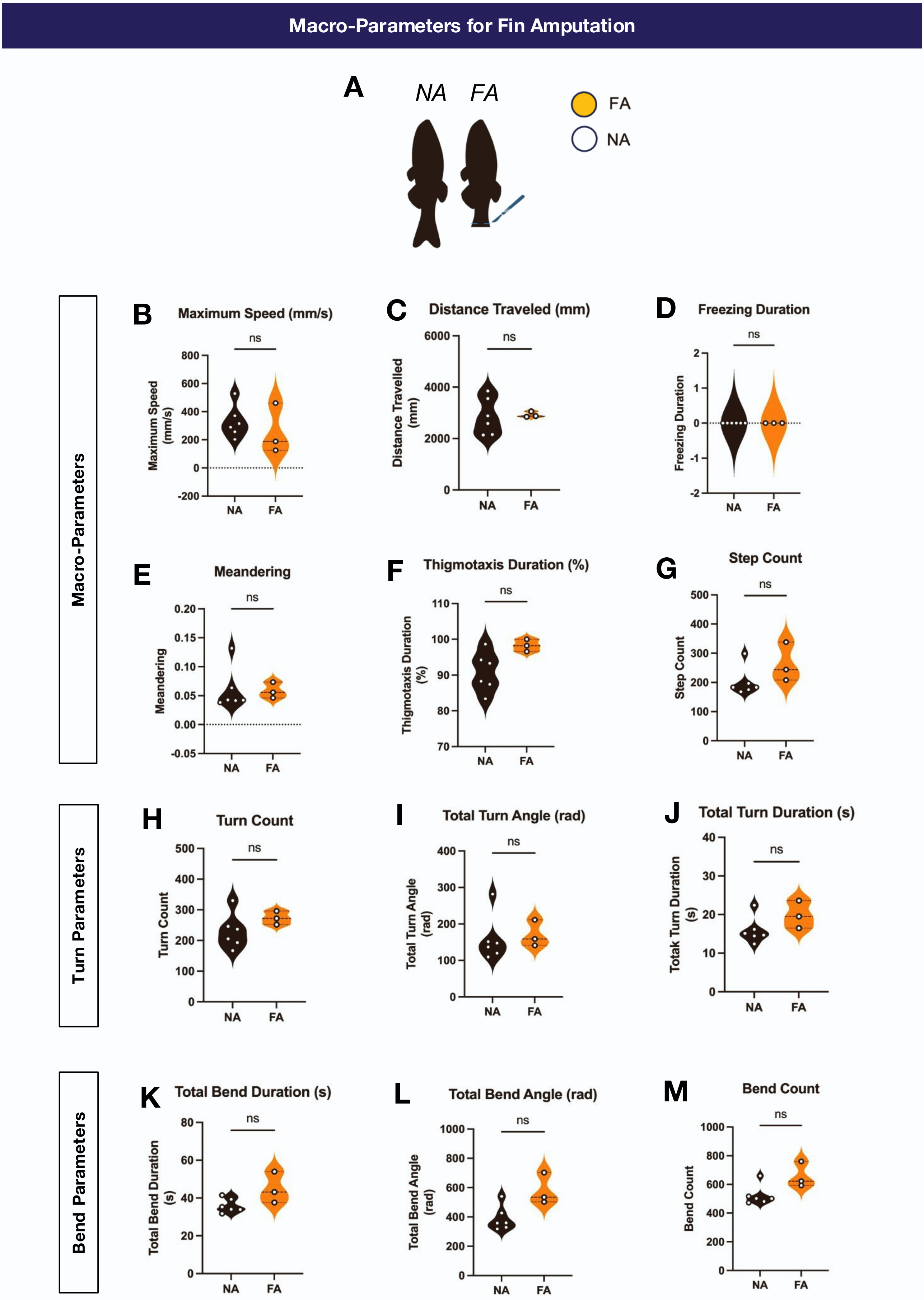

Supplementary Figure 8. No significant differences in locomotion macro-parameters were observed in fin amputated (FA) compared to non-amputated (NA) zebrafish. (A) An illustration showing comparisons between non-amputated (NA) and fin amputated (FA) zebrafish adults. No significant differences between FA and NA was observed in the following parameters (B) maximum speed, (C) distance travelled, (D) freezing duration, (E) meandering, (F) thigmotaxis duration, (G) step count, (H) turn count, (I) total turn angle, (J) total turn duration, (K) total bend duration, (L) total bend angle, nor (M) bend count. Data was analyzed for statistical significance using nested t-test, unless otherwise stated. All data are presented as mean ± SEM, where no significance was shown (FA) = 3, and n (NA) = 6.

Supplementary Figure 9

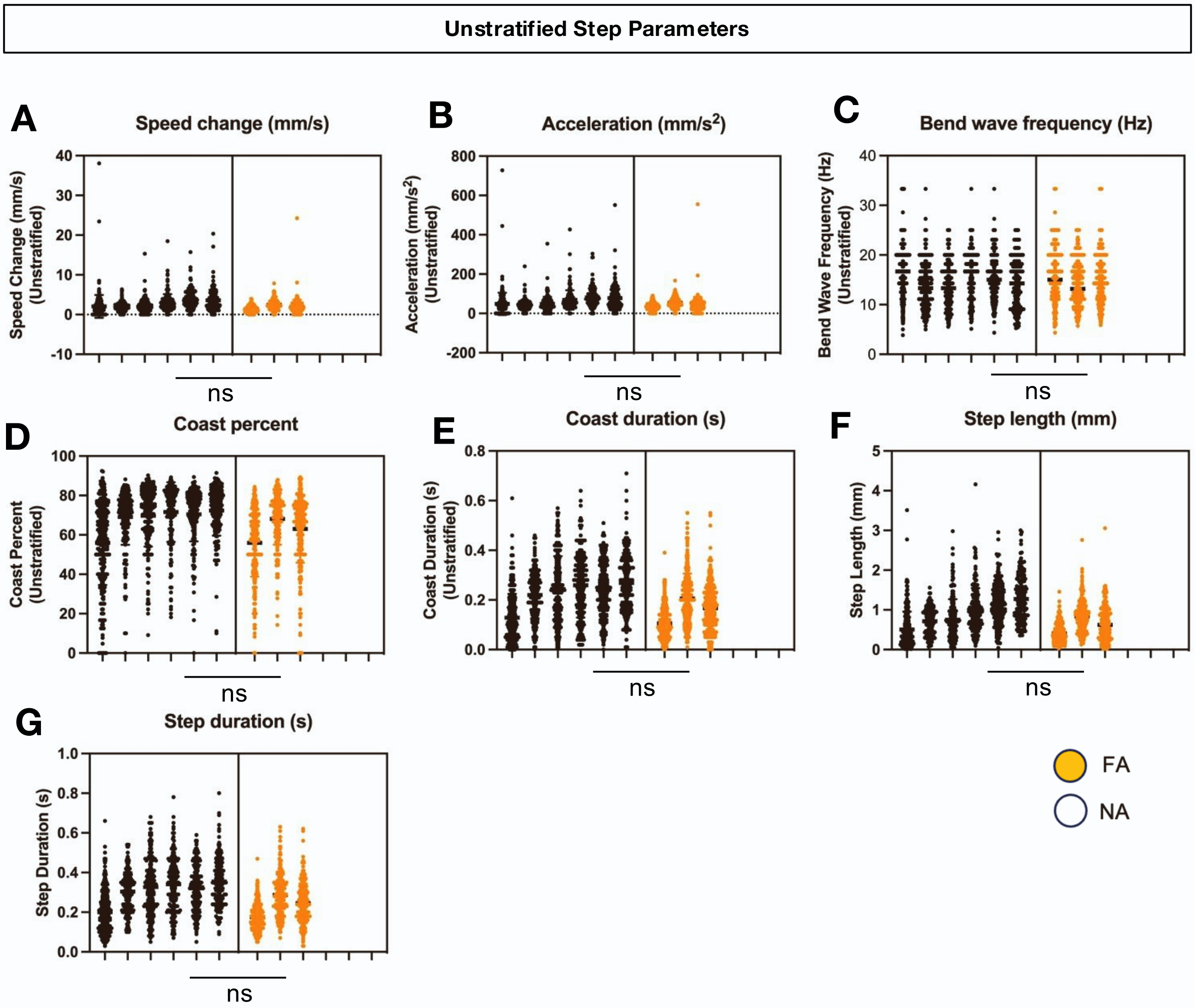

Supplementary Figure 9. No significant difference was observed in unstratified step parameters in fin amputated (FA) zebrafish compared to non-amputated (NA) controls. No significant differences between FA and NA zebrafish were detected in **(A)** swimming speed, **(B)** acceleration, **(C)** bend wave frequency, **(D)** coast percent, **(E)** coast duration, **(F)** step length, and **(G)** step duration. Data was analyzed for statistical significance using nested t-test, unless otherwise stated. All data are presented as mean ± SEM, where no significance was shown (FA) = 3, and n (NA) = 6.

Supplementary Figure 10

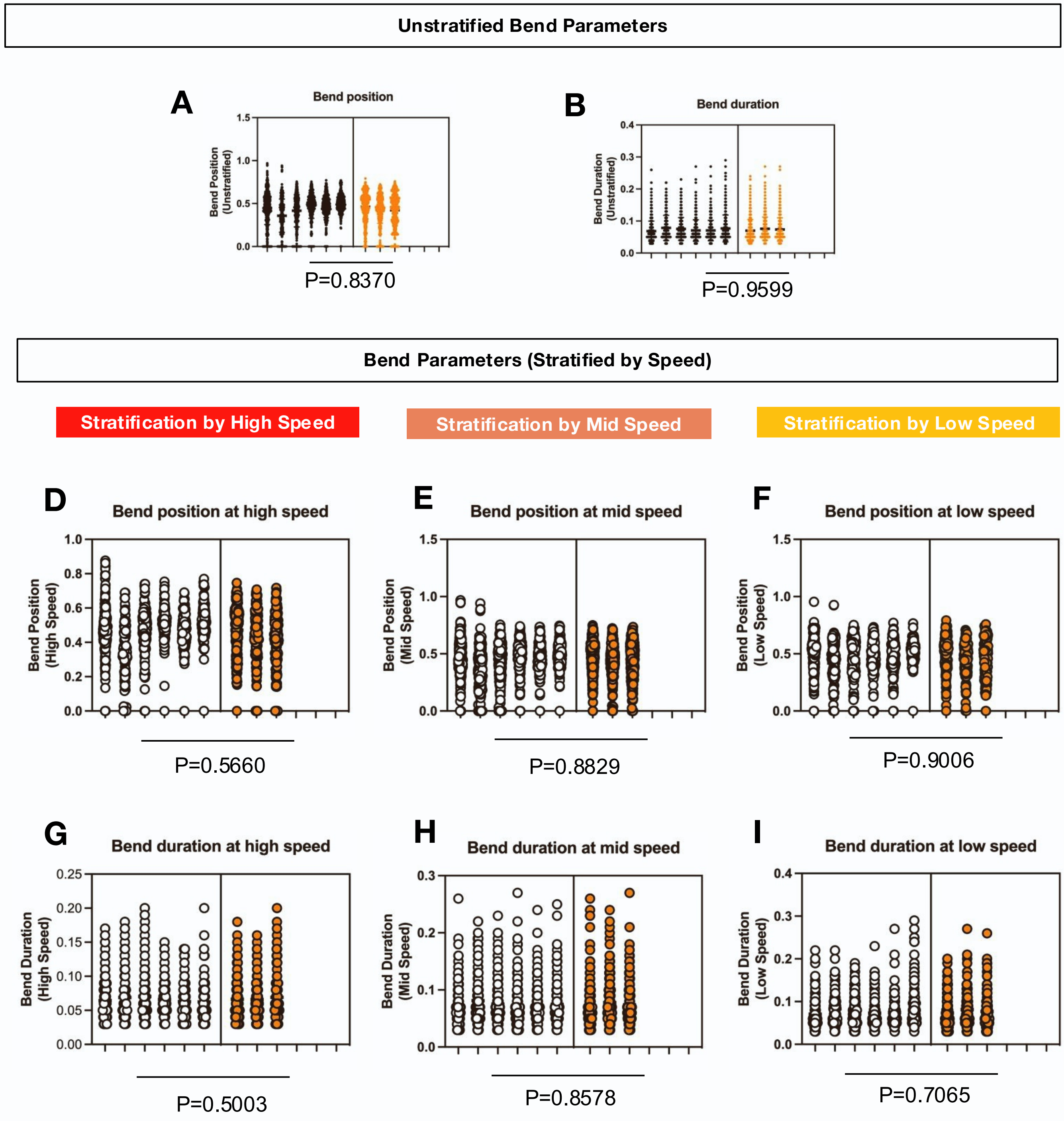

Supplementary Figure 10. No significant difference observed in unstratified and stratified 'bend' parameters when comparing between fin amputated (FA) zebrafish and non-amputated (NA) controls. No significant differences in unstratified **(A)** bend position, and **(B)** bend duration between FA and NA. When stratified against speed, there were still no significant differences in the bend position between FA and NA during **(D)** high, **(E)** mid, and **(F)** low speeds. When stratified against speed, there were still not significant differences in the bend duration between FA and NA during **(G)** high, **(H)** mid, and **(I)** low speeds. Data was analyzed for statistical significance using nested t-test, unless otherwise stated. All data are presented as mean ± SEM, where no significance was shown (FA) = 3, and n (NA) = 6.

Supplementary Figure 11

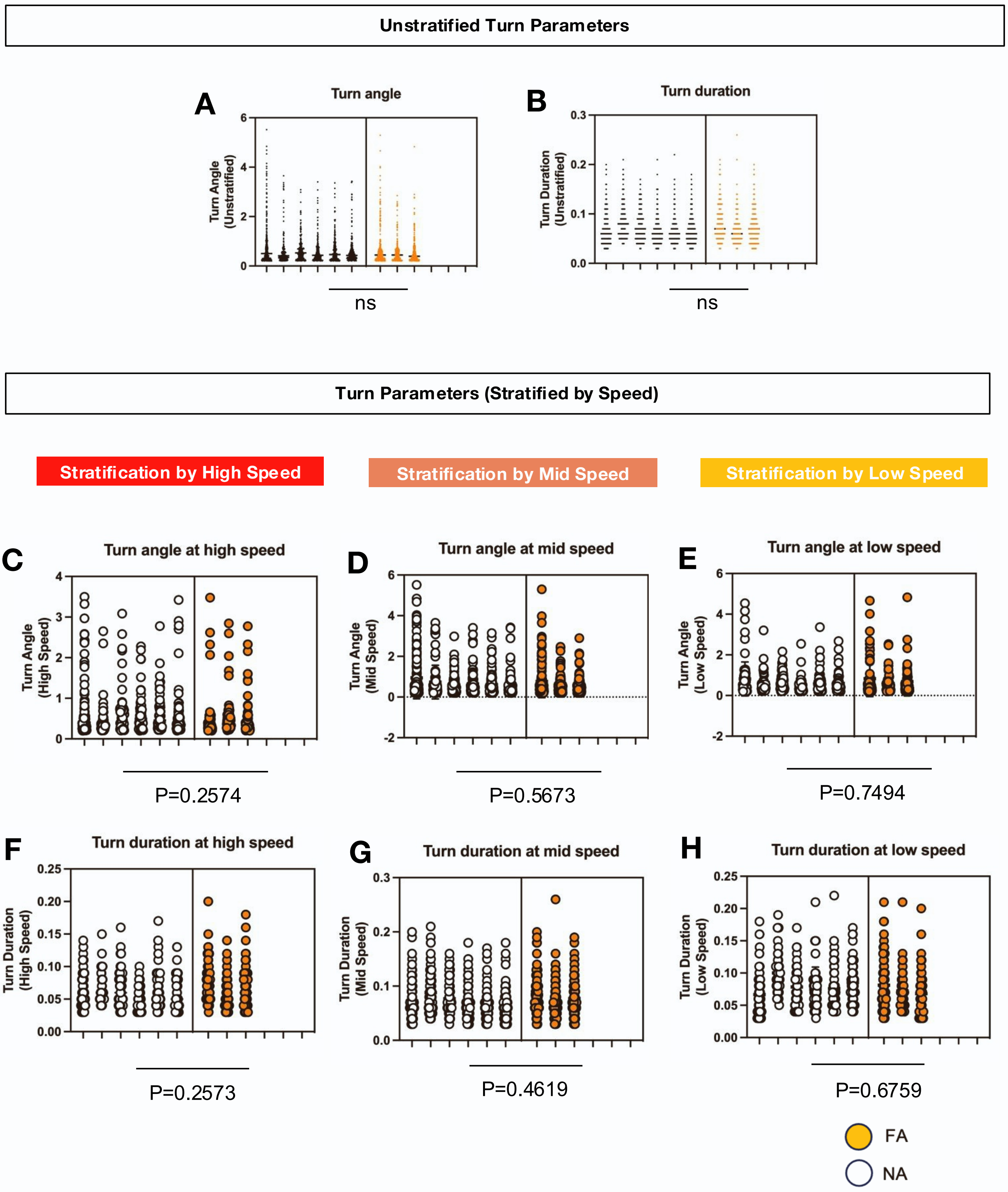

Supplementary Figure 11. No significant difference observed in unstratified and stratified 'turn' parameters when comparing between fin amputated (FA) zebrafish and non-amputated (NA) controls. No significant differences in unstratified **(A)** turn angle, nor (B) Turn duration was observed comparing between FA and NA. When stratified against speed, there were still no significant differences in the turn angle between FA and NA during **(C)** high, **(D)** mid, and **(E)** low speeds. When stratified against speed, there were still no significant differences in the turn duration between FA and NA during **(F)** high, **(G)** mid, and **(H)** low speeds. Data was analyzed for statistical significance using nested t-test, unless otherwise stated. All data are presented as mean ± SEM, where no significance was shown (FA) = 3, and n (NA) = 6.
